# Fast Detection of 2,4,6-Trinitrotoluene (TNT) at ppt Level by a Laser-Induced Immunofluorometric Biosensor

**DOI:** 10.3390/bios10080089

**Published:** 2020-08-05

**Authors:** Martin Paul, Georg Tscheuschner, Stefan Herrmann, Michael G. Weller

**Affiliations:** Division 1.5 Protein Analysis, Federal Institute for Materials Research and Testing (BAM), Richard-Willstätter-Strasse 11, 12489 Berlin, Germany; martin.paul@bam.de (M.P.); georg.tscheuschner@bam.de (G.T.); sth1812@gmx.de (S.H.)

**Keywords:** aviation security, biosensor, flow injection assay, monoclonal antibody, fluorescence microscope, lab-on-a-chip, microfluidic systems, antibody labeling, CMOS, diode laser, monolithic column, laser-induced fluorescence detector (LIF), additive manufacturing, low-cost, high-speed, non-competitive immunoassay, immunometric assay

## Abstract

The illegal use of explosives by terrorists and other criminals is an increasing issue in public spaces, such as airports, railway stations, highways, sports venues, theaters, and other large buildings. Security in these environments can be achieved by different means, including the installation of scanners and other analytical devices to detect ultra-small traces of explosives in a very short time-frame to be able to take action as early as possible to prevent the detonation of such devices. Unfortunately, an ideal explosive detection system still does not exist, which means that a compromise is needed in practice. Most detection devices lack the extreme analytical sensitivity, which is nevertheless necessary due to the low vapor pressure of nearly all explosives. In addition, the rate of false positives needs to be virtually zero, which is also very difficult to achieve. Here we present an immunosensor system based on kinetic competition, which is known to be very fast and may even overcome affinity limitation, which impairs the performance of many traditional competitive assays. This immunosensor consists of a monolithic glass column with a vast excess of immobilized hapten, which traps the fluorescently labeled antibody as long as no explosive is present. In the case of the explosive 2,4,6-trinitrotoluene (TNT), some binding sites of the antibody will be blocked, which leads to an immediate breakthrough of the labeled protein, detectable by highly sensitive laser-induced fluorescence with the help of a Peltier-cooled complementary metal-oxide-semiconductor (CMOS) camera. Liquid handling is performed with high-precision syringe pumps and chip-based mixing-devices and flow-cells. The system achieved limits of detection of 1 pM (1 ppt) of the fluorescent label and around 100 pM (20 ppt) of TNT. The total assay time is less than 8 min. A cross-reactivity test with 5000 pM solutions showed no signal by pentaerythritol tetranitrate (PETN), 1,3,5-trinitroperhydro-1,3,5-triazine (RDX), and octahydro-1,3,5,7-tetranitro-1,3,5,7-tetrazocine (HMX). This immunosensor belongs to the most sensitive and fastest detectors for TNT with no significant cross-reactivity by non-related compounds. The consumption of the labeled antibody is surprisingly low: 1 mg of the reagent would be sufficient for more than one year of continuous biosensor operation.

## 1. Introduction

The fast and extremely sensitive detection of explosives [1,2,3] is one of the most relevant tasks to guarantee security in areas of public access. Many airplane passengers are confronted with some security measures, from which the ban of most liquids in the luggage is one of the least popular. X-ray-based scanners are used in most airports, which may detect larger amounts of explosives. However, for trace analysis, additional wipe tests may be necessary. Nevertheless, the ultimate explosive detector is still the dog, which is requested in nearly all critical situations. Considering the high cost of a trained animal with its handler and the inability to use them for extended missions, it becomes clear that an automated sensor system would be highly desirable. The first steps towards an electronic dog nose were published some time ago [4]. Unfortunately, no sensor system can compete with dogs and other animals, yet. In addition, the most powerful instrumental analysis systems are not mobile and are limited to a laboratory environment.

Up to now, several sensor systems have been developed to detect traces of explosives. Perhaps the most well-known devices are based on ion mobility spectrometry (IMS) [5], which are commercially available and claim sensitivities down to ppb. However, sensitivity and particularly selectivity still need to be improved [6]; hence false positives from household products seem to be common. Quite a few chemosensors have been presented [7], which are often based on quartz microbalances [8,9] or fluorescence quenching, e.g., [10,11,12,13,14,15,16,17,18]. Many sensors of the latter type display stunning sensitivity, which may explain their popularity in the research field. Unfortunately, most publications show only very sparse cross-reactivity data. In addition, the transfer of these systems to other explosives, such as pentaerythritol tetranitrate (PETN) or triacetone triperoxide (TATP), seems to be generally difficult, if not impossible.

A review of different luminescence-based methods was published in 2008 [19]. In the same year, a review of biosensors and bioinspired systems appeared [20]. In this article, not only antibody-based methods were mentioned, but also systems using aptamers, peptides [21], cyclodextrins, molecularly imprinted polymers (MIPs) [22,23,24], odorant-binding proteins, bacteria, algae, and yeasts. A particularly interesting concept is the combination of MIPs with fluorescence, which combines the selectivity of MIPs with the sensitivity of fluorescence detection [25]. Furthermore, immunosensors have been examined for a while [26,27,28,29,30,31,32,33,34,35,36]. Some of them are based on surface plasmon resonance technology (SPR) [37,38]. Others used antibody-gated mesoporous silica nanoparticles [39]. Also, electrochemical immunosensors have been developed [40,41]. They often showed promising performance parameters. Unfortunately, not many antibodies against explosives are available, although some had been developed and characterized [42,43,44,45,46,47,48,49,50,51,52]. However, their broader development seems to have stalled for some time.

For the fast and sensitive detection of explosives with antibodies, immunometric assays are promising. This concept was presented by Freytag et al. in 1984 [53,54,55]. Later these assay types were varied and discussed in more detail [56,57,58,59,60,61,62,63,64,65,66]. Unfortunately, the terminology for these assays is quite diverse. To the best of our knowledge, this approach has never been used for security applications before. A somehow related format is known as the kinetic exclusion assay [67,68] which, however, detects the bound receptor molecules and not the eluted fraction.

**Figure 1:**
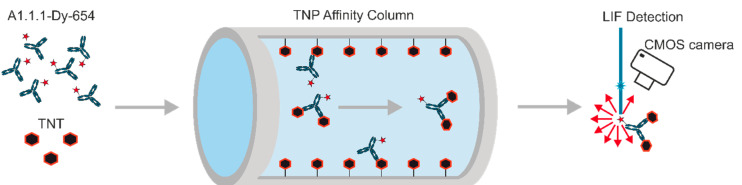
A biosensor based on an immunofluorometric assay: The monoclonal antibody A1.1.1 against 2,4,6-trinitrotoluene (TNT) is labeled with the fluorescent dye Dy-654. This conjugate is briefly preincubated with the sample and pumped through the antigen-coated affinity column, which traps free antibody. Only antibody-TNT complexes reach the highly sensitive, laser-induced fluorescence (LIF) detector.

In our format, a labeled high-affinity antibody is mixed, preincubated with the sample, and passed through a hapten-coated affinity column. A fluorescent antibody with free binding sites can bind to the affinity column. In contrast, antibodies with binding sites blocked by analyte molecules would not be retained, instantly elute from the column, and are detected in a sensitive fluorescence detector (Figure 1).

As antibody binding is a reversible process, antibodies with off-rates far longer than the column passage time are required to allow sensitive detection of the analyte. This means that high-affinity antibodies are needed for optimal assay performance. Furthermore, inactive antibodies would contribute to the background of this assay. Hence, highly purified antibody conjugates are preferable.

## 2. Materials and Methods

### 2.1. Reagents, Buffers, Materials, and Equipment

Transparent, flat-bottom high binding 96-well microtiter plates (655101) were acquired from Greiner Bio-One (Frickenhausen, Germany), PD SpinTrap™ G-25 Desalting Columns (28918004) were obtained from GE Healthcare (Uppsala, Sweden), monoclonal anti-2,4,6-trinitrotoluene (TNT) antibody A1.1.1 (mouse, subtype IgG_1_) [45] was obtained from SDIX (Newark, USA), goat anti-mouse horseradish peroxidase-(HRP)-conjugated antibody (15–035–003) was obtained from Jackson immune research (Cambridge, UK), fluorescence dye Dy-654-NHS and Dy-654-COOH were purchased from Dyomics (Jena, Germany). According to the manufacturer, the following properties of the fluorescent dye Dy-654 are given: excitation/emission max. 653/677 nm (in ethanol), molar absorbance: 220.000 M^−1^cm^−1^, soluble in water, methanol, and DMF (https://dyomics.com/en/products/red-excitation/dy-654). Bovine serum albumin (BSA) >98% (A7906), diethoxy(3-glycidyloxypropyl)-methyl silane (539252), Mucasol (Z637203), ProClin300 (8912-U) and 5% (w/v) picrylsulfonic acid solution (TNBS, P2297) were purchased from Sigma-Aldrich (Taufkirchen, Germany). Hydrochloric acid (HCl, 84415) was purchased from Fluka, and cyano-4-hydroxycinnamic acid (CHCA) was bought from Bruker Daltonics (Bremen, Germany), sodium bicarbonate (1940) and potassium hydroxide (121515) were obtained from AppliChem (Darmstadt, Germany), tetramethylbenzidine (TMB) substrate (SeramunBlau fast2) were bought from Seramun (Heidesee, Germany), Tween 20 (37470.01) was bought from Serva (Heidelberg, Germany), absolute ethanol (2246) from Th. Geyer (Renningen, Germany) and labeling grade DMF (13050) was bought from Lumiprobe (Hunt Valley, USA). TNT, PETN, 1,3,5-trinitroperhydro-1,3,5-triazine (RDX), octahydro-1,3,5,7-tetranitro-1,3,5,7-tetrazocine (HMX) were kindly supplied by BAM Division 2.3. Vitrapor5 glass monoliths were acquired from ROBU (Hattert, Germany), and ultrapure water (MilliQ) was supplied by a Milli-Q Synthesis A10 system (Merck, Germany). The bandpass filter (FL635–10), the dichroic mirror (DMLP638R), the long pass filters (FELH650), and the tube lens (TTL165-A) were purchased from Thorlabs (Newton, USA). The objective plan achromat 10×/0.25 Ph1 (415500–1605–001) was obtained from Carl Zeiss (Oberkochen, Germany), the diode laser 70105582 from Picotronic (Koblenz, Germany), the microfluidic chip (10000091) from Microfluidic ChipShop (Jena, Germany), the injection valve (5067–4158) from Agilent (Santa Clara, USA) and a Fusion 4000 syringe pump was acquired from Chemyx (Stafford, USA). Matrix-assisted laser desorption/ionization time-of-flight (MALDI-TOF) mass spectrometry was performed on a Bruker Autoflex II Smartbeam, and Autoflex Max MS and absorbance was measured with an Epoch2 photo spectrometer from Biotek (Vermont, USA). Data evaluation was performed with Python 3.7 in Anaconda (Spyder 3.3.2) and Origin (2018G). 

### 2.2. Trinitrophenyl-BSA Conjugates and Indirect Competitive Enzyme-Linked Immunosorbent Assay (ELISA)

Trinitrophenyl-(TNP)-BSA conjugate for the affinity column coating: 20 mg (0.3 µmol) of BSA was dissolved in 1 mL of 0.2 M NaHCO_3_, and 26.4 µL (4.5 µmol) of 5 (w/v)% aqueous trinitrobenzene sulfonic acid (TNBS) was added, vortexed and stored for one hour at RT and subsequently for 48 h at 4 °C. After incubation, 125 µL of 2 M NaH_2_PO_4_ was added to adjust the solution to a neutral pH. A mean degree of labeling (DOL) of approximately 8 TNP per BSA was determined with MALDI-TOF MS (see Figure S2)

TNP-BSA conjugate for indirect enzyme-linked immunosorbent assay (ELISA): In 5 mL of 0.2 M NaHCO_3_ 100 mg (1.5 µmol) of BSA were dissolved and 88 µL (15 µmol) of TNBS (5%) were added, vortexed and stored for 1 h at RT and subsequently for 48 h at 4 °C. After incubation, 625 µL of 2 M NaH_2_PO_4_ was added to adjust the solution to a neutral pH. A DOL of 5 TNP per BSA was determined by MALDI-TOF MS (see Figure S14).

ELISA procedure: Clear, high binding 96-well plates (MTP) with flat bottom were coated with 100 µL of a blend of 0.023 g/L TNP-BSA and 0.75 g/L BSA in phosphate-buffered saline (PBS) with 10 mM phosphate and 137 mM sodium chloride pH = 7.4 (100 µL per well). The plate was sealed with Parafilm, protected from light with aluminum foil and shaken at 750 rpm for 20 h at RT. The MTP was washed with PBS containing 0.05 vol% Tween 20 by an automated plate washer three times.

Then 50 µL of diluted TNT in PBS ranging from 10 pM to 10 µM and 50 µL of 1:20,000 diluted A1.1.1-Dy-654 (approximately 17.5 µg/L) in PBS were added as quadruplicates and incubated for one hour at RT in the dark.

After a washing step, 100 µL of 40 µg/L HRP-conjugated anti-mouse (H + L) IgG antibody in PBS with 1% BSA were incubated for one hour in the dark and the MTP was subsequently washed. Then 100 µL TMB substrate (Seramun Blau fast2) was incubated for 30 min and stopped with 100 µL of 0.25 M sulfuric acid. The absorbance was recorded with an Epoch2 Photometer at 450 and 620 nm. 

### 2.3. Manufacturing of Affinity Columns 

Cylindrical Vitrapor5 glass monoliths (15 × 8 mm) were glued into titanium shells (15 × 12 mm, wall thickness: 1 mm) with silicone glue and inserted into custom-designed and additively manufactured column holders with matching 1/16” threaded polyether ether ketone (PEEK) inlets (see Figure S1, Supplementary Materials).

The column was cleaned, and silanized with diethoxy(3-glycidyloxypropyl)-methyl silane, as described in Table S1 (Supplementary Materials). 

For the preparation of the TNP-BSA affinity column, 440 µL of the 7.8 eq. TNP-BSA solution was diluted with 2100 µL 0.1 M Na_2_HPO_4_ pH 8.1 and incubated for one week at RT on the epoxy-functionalized raw column. 

### 2.4. Design and Synthesis of the A1.1.1 Fluorophore Conjugate

In order to prepare the 4.54 mM Dy-654-NHS labeling solution, 0.2 mg of the NHS ester (0.18 µM) were dissolved in 40 µL of dry, amine-free DMF (labeling grade, Lumiprobe) and aliquoted to 10 µL portions and stored in the dark at −18 °C.

For antibody labeling, 9.18 µL (0.68 nmol) of the A1.1.1 stock solution (10.9 g/L) in PBS containing 0.05 wt.% NaN_3_ was diluted with 91 µL of PBS to 1 mg/L. A PD Spintrap G-25 was centrifuged dry for one minute at 800 g and 4 °C and subsequently purged and centrifuged four times with 140 µL of 0.1 M Na_2_HPO_4_ and 1.37 M NaCl adjusted to pH 7.8. On the conditioned PD Spintrap, 100 µL of diluted antibody-solution and an additional 40 µL stacker volume of PBS were transferred and centrifuged at 800× *g* and 4 °C for one minute, 140 µL of eluate were collected. The eluate, containing approximately 95 µg A1.1.1 (0.63 nmol IgG), was cooled to 4 °C and six-fold access of the label, 0.84 µL of the labeling solution (3.8 nmol Dy-654-NHS) were added. The solutions were gently mixed by pipetting and incubated for 3 h at 14 °C in the dark and for 18 h in the dark at 4 °C. The solution was purified with a PBS conditioned PD Spintrap G-25. The conjugate was stabilized with 0.04 vol% ProClin300 and stored in the dark at 4 °C.

### 2.5. Fluorescence Detector

The custom detector based on an epifluorescence microscope setup was built from modular and affordable parts (see Table S2, Supplementary Materials). The detailed construction plans can be found in Figure S4 (SI). As a detector, the camera QHY174M-GPS from QHYCCD (Beijing, China) was used, featuring an IMX-174M (Sony) CMOS sensor. This sensor is thermoelectrically (Peltier) cooled, has 5.86 × 5.86 µm square pixels, and delivers a maximal dynamic range of 12 bit. Data are acquired via a USB 3.0 connection by a laptop with the Software SharpCap (Version 3.0.4074.0). 

**Figure 2:**
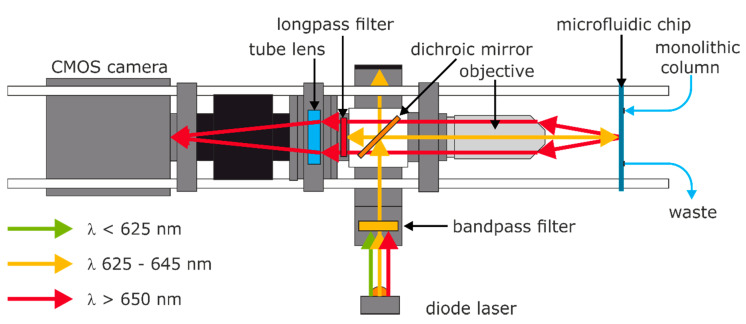
Light paths in the setup with essential optical components.

In the excitation path (Figure 2, orange color), a diode laser (70105582, Picotronic) with a measured center-wavelength of 638 nm with a full width at half maximum (FWHM) of approximately 3 nm (see Figure S7), and an optical output power below 1 mW was used. The laser was directed through an FL635–10 bandpass filter and guided at 45° on a dichroic mirror (DMLP638R, THORLABS) with a cutoff wavelength of 638 nm; the excitation was focused by an infinity-corrected plan achromat 10×/0.25 Ph1 objective (415500–1605–001, ZEISS) on a microfluidic COC (cyclic olefin copolymer) chip. The microfluidic chip (10000091, Microfluidic ChipShop) features four 200 × 200 µm linear flow channels and is mounted on a custom holder for precise adjustment (see Figure S6). In the fluorescence path (Figure 2, red color), the fluorescence of the label in the flow channel is collected by the objective, filtered by two stacked long-pass filters (FELH650, THORLABS), and focused with tube lens (focal length 165 mm, TTl165-A, THORLABS) on the sensor of the camera (QHY174M-GPS, QHY). The whole setup is mounted on an optical breadboard and protected from environmental light and dust with a box made of black cardboard.

### 2.6. Measurements

All samples and buffers were injected with a Fusion 4000 dual syringe pump (Chemyx). The flow was directed to the column holder, which contained the monolithic column, and the eluate was subsequently passed through the flow channel of the microfluidic chip (see Figure 2). In order to ensure bioinert conditions, all connectors were manufactured from PEEK or PP, and the seals were made from silicone. The column holder was made by additive manufacturing and had custom PEEK connectors; the columns may conveniently be exchanged (see Figure S1). The typical backpressure of the monolithic column at a flow rate of 0.5 mL min^−1^ was approximately 2.7 bar (see Figure S3). Therefore, the actual overall system pressure is low enough to operate the whole fluidics with standard plastic (PP) syringes and PTFE-silicone tubes. The total flow rates were limited to 0.5 mL min^−1^ to protect the microfluidic chip with the linear flow cell.

Setup of the sensitivity test: Dy-654-COOH was diluted to from 5 to 25 pM in 5 pM steps and from 100 to 1000 pM in 100 pM steps and injected with a 12 mL syringe with PBS blanks in between the samples. The data were recorded with an exposure time of 5000 ms; the gain was set to four, and the sensor temperature to −5 °C. 

**Figure 3:**
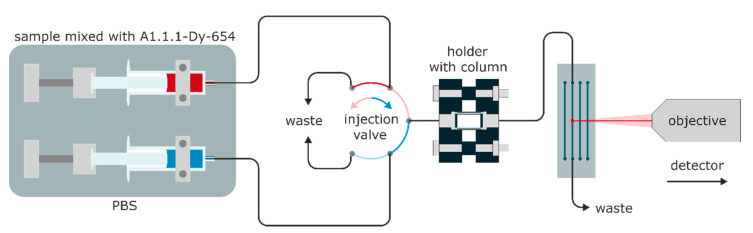
Microfluidic setup for the detection of TNT and the cross-reactivity (CR) determination with six-way-valve for continuous flow injection.

TNT detection: TNT was diluted from a stock solution in ethanol to 0.4 to 2 nM in 0.4 nM steps and from 4 to 20 nM in 4 nM steps in PBS with 0.1 (w/v)% BSA and mixed 1:1 with A1.1.1-Dy-654 (1:50,000, approximately 7 µg/L) in PBS with 0.1 (w/v)% BSA, incubated for five minutes and injected through a six-way-valve as shown in Figure 3. The data were recorded as described above.

Cross-reactivity tests: TNT, PETN, RDX, and HMX were diluted from a stock solution in methanol to 10 nM in PBS with 0.1 (w/v)% BSA and mixed 1:1 with diluted A1.1.1-Dy-654 (1:50,000, approximately 7 µg/L) in PBS with 0.1 (w/v)% BSA, incubated for five minutes and injected at a flow rate of 0.5 mL/min with six-way-valve as shown in Figure 3. The data were recorded as described above. 

## 3. Results

### 3.1. Fluorescence Detector

The two stacked long-pass filters and the dichroic mirror efficiently prevent stray light from reaching the CMOS sensor. The excitation laser has a center wavelength of 638 nm and an FWHM of approximately 3 nm. In preliminary experiments, the use of an additional second long-pass filter has proven to be beneficial. The microfluidic chip is made from TOPAS^®^ (cyclic olefin copolymer, COC), which shows a very low autofluorescence at the used wavelength and has a smooth flat and transparent surface suitable for observation. The fluorescence is collected with the same 10X0.25 NA objective and guided through the dichroic mirror and passed through the long-pass-filters. Overall a high transmission for the fluorescence is to be expected as the filters show high transmission of about 90% at 654 nm, which is the peak wavelength of Dy-654 emission. The monochromatic sensor of the QHY is reported to exhibit a high quantum yield of approximately 50% at 650 nm and is, therefore, well-suited for the application. The position of the excitation laser spot on the microfluidic chip can be conveniently adjusted by the laser holder (see Figure S5) and is set in the center of the flow channel. 

### 3.2. Semi-Automated Data Evaluation with Python

The images are recorded as a sequence of raw files (.fits) and have a native resolution of 1920 × 1080 pixels. The size of the laser-illuminated spot, which essentially defines the region of interest (ROI), is only a few pixels wide. Due to this small area, the exact position of the ROI in every frame (exposure time: 5000 ms) is of high importance for the correct and reliable data evaluation. It was observed that within elongated measurement periods, the ROI position might be shifted slightly by a few pixels in x- and or y-direction. This behavior is most likely a result of the thermal expansion of the additive manufactured laser-holder (Figure S5). In order to account for this shift, the correct position of the ROI must be determined automatically but precisely for every frame. By a semi-automated python-script, a Gaussian fit is utilized to determine the center pixel of the laser spot, based on which a pixel-square ROI is defined.

Around the determined laser center, a square ROI region of 8 × 8 pixels is defined, and all 64 pixels inside are sorted by their intensity. The three most intense pixels are discarded to account for possible cosmic rays or hot pixels. Subsequently, the following five pixels are used to calculate the mean value, which is defined as intensity for the frame. To determine the signal intensity of an injected probe, for example, the 100 pM Dy-654-COOH solution, 16 frames are used to calculate the mean value and the standard deviation. To determine the starting point for the signal evaluation (f_n_), the raw-signal is smoothed by a Savitzky–Golay filter with a polynomial of second-order and a window of five frames. Subsequently, the gradient of the smoothed data is calculated. The first sample point of the 1st derivative to fall below zero after the initial signal increase is picked (see Figure S8) and defined as f_n_, as it represents a stable signal as growth is completed. Based on this frame, f_n_ and the next 15 frames are used to calculate the mean and the standard deviation of the signal.

### 3.3. Antibody Selection, Validation, Labeling, and Determination of the Test Midpoint for 2,4,6-Trinitrotoluene (TNT)

The employed antibody ultimately governs the quality of an immunoassay. Thus, the antibody used must be chosen carefully with sensitivity, selectivity, and stability in mind. Many manufacturers only give an order number of an antibody. The properties or even the true clone identity may remain unclear [69]. This creates a risk for reliable and reproducible assays, which must not be tolerated when this information is critical. Two commercially available monoclonal anti-TNT antibodies A1.1.1 (SDIX, USA) and EW75C (BBI Solutions, UK) from mouse IgG_1_ subclass were evaluated for their affinity to TNT for validation purposes with indirect competitive ELISA as described in Section 2.2. Also, their specific cross-reactivities with compounds of interest and high explosives (see Table S2 and Figures S16 and S17) were determined. The clone A1.1.1 showed superior affinity to the analyte TNT by a factor of 30 (Figure S18) compared to the clone EW75C and was, therefore, chosen for this project. Additionally, for both clones, a mass spectrometric antibody fingerprint, according to Tscheuschner et al. [69], was generated (see Figure S19), which may be useful to identify these clones in future work. 

**Figure 4:**
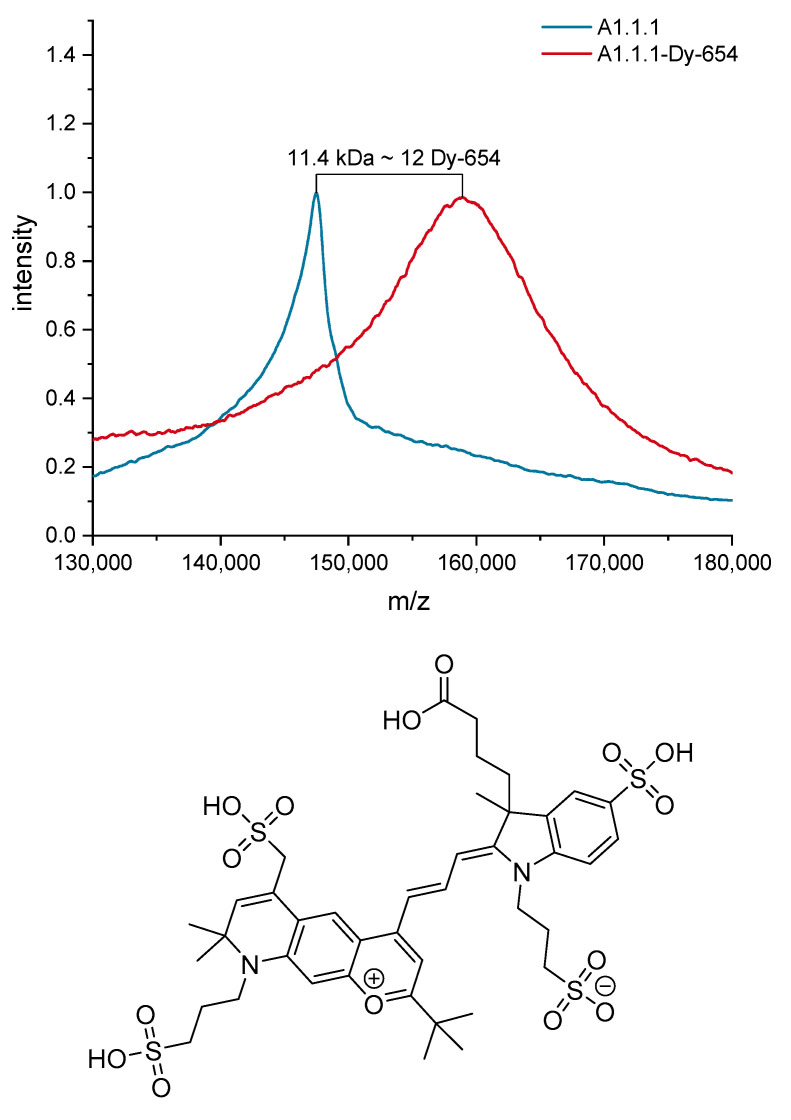
Smoothed MALDI-TOF MS spectra of the antibody A1.1.1 and the conjugate with the fluorescent dye Dy-654 (top) and structure of the label Dy-654-COOH (MW 1007.08 g/mol, Dyomics).

The sensor system relies on the sensitive detection of fluorophore-labeled antibodies in the eluate of the affinity column. A proper choice of fluorophore is, therefore, of considerable importance. A suitable label should display desirable properties like high quantum yield, high photostability, excellent water solubility, reduced aggregation, and low non-specific binding. Additionally, no detectable cross-reactivity with the antibody is imperative. Based on the available laser excitation source of 638 nm and excellent performance on epoxy-functionalized glass substrates [70], the cyanine dye Dy-654 [Figure 4] was chosen. The label features four sulfonic acids, which results in highly hydrophilic properties and a minimal tendency for aggregation. Furthermore, the dye showed no detectable cross-reactivity with the antibody A1.1.1 in preliminary experiments.

**Figure 5:**
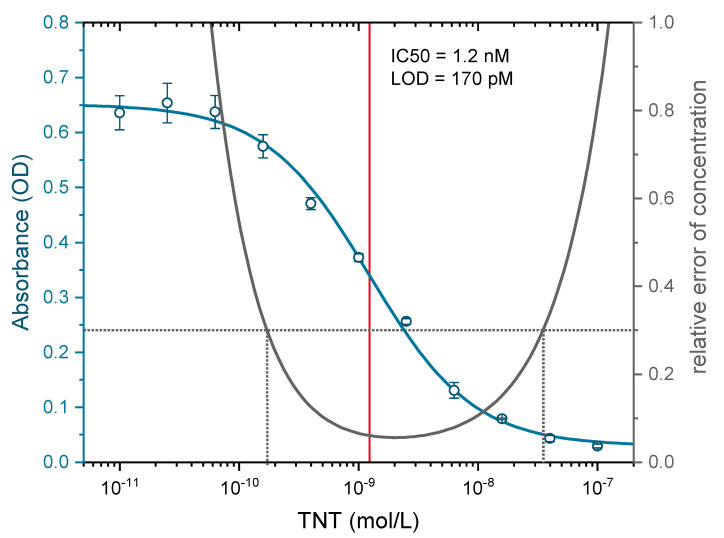
Calibration curve (blue) and precision profile (black) of an indirect enzyme-linked immunosorbent assay (ELISA) with the monoclonal antibody A1.1.1. The analyte TNT was determined in quadruplicates.

The degree of labeling (DOL) for the A1.1.1-Dy-654 conjugate was determined with MALDI-TOF MS to be approximately 12 (see Figure 4) and the protein concentration of the A1.1.1-Dy-654 stock solution was determined to contain approximately 0.35 g/L antibody according to UV measurements. The test midpoint (IC_50_) was determined by indirect competitive ELISA to be 1.2 nM (see Figure 5), which is in excellent agreement with the literature stated value determined for the clone A1.1.1 of 1.3 nM [45]. The limit of detection (LOD) of the indirect ELISA was determined to be approximately 170 pM.

### 3.4. 2,4,6-Trinitrophenyl-(TNP)-BSA Affinity Columns 

The degree of labeling of the BSA was determined by MALDI-TOF MS to be approximately 8 TNP molecules per BSA (see Figure S2, Supplementary Materials). In preliminary tests, the TNP-BSA and the BSA column showed no non-specific interaction for the label Dy-654-COOH or Dy-654-labeled human IgG (Avastin, Bevacizumab). The backpressure of a monolithic column was determined to be about 2.7 bar at a flow rate of 0.5 mL min ^−1^ of PBS (see Figure S3). The columns were purged with a mixture of 80 vol% ethanol/water and stored immersed in this solution in a sealed vial at 4 °C for several months without noticeable degradation of column performance.

### 3.5. Setup Optimization and Limit of Detection (LOD) of the Label

The exposure time, the sensor gain, and the sensor temperature were varied to determine the optimal ratio of signal height and noise (S/N). To calculate the S/N, the signal difference between the signal intensity of 100 pM Dy-654-COOH dissolved in PBS and pure PBS was divided by the sum of the standard deviation of the Dy-654-COOH and the blank signal. The most substantial influence on the S/N was observed for the exposure time. Longer exposure times up to 5000 ms and even beyond, increased the S/N (see Figure S9). Increasing sensor gain reduced the S/N, especially at a gain >4 (see Figure S10). The temperature had no clear impact on S/N (see Figure S11), as the known hot pixels were already removed by the python script. An exposure time of 5000 ms and a gain of 4 was chosen to achieve a wide dynamic range and acceptable response times. The sensor temperature was set to –5 °C, which was the lowest temperature the camera was able to keep over longer times at ambient temperatures around 25 °C. These settings were applied in all measurements in this paper if not stated otherwise.

**Figure 6:**
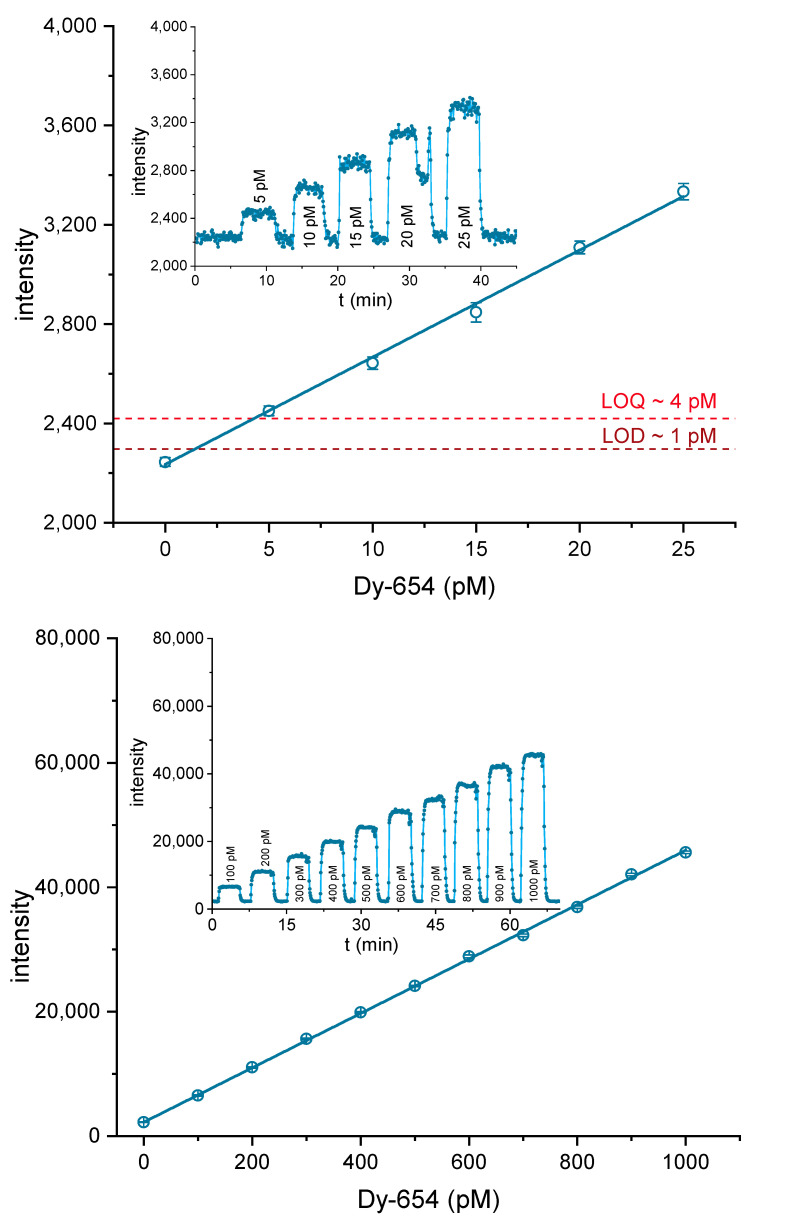
Determination of the limit of detection (LOD) (3s) and LOQ (10s) for the fluorescence label Dy-654-COOH (top) and calibration line in the linear range from 4–1000 pM (bottom).

Solutions of Dy-654-COOH were prepared in PBS from 5 to 25 pM to determine the LOD and limit of quantification (LOQ) for the label and dilutions from 100 to 1000 pM to assess the range of the linear response (see Figure 6, top). After a stable signal was achieved, 16 frames were used to calculate the mean and standard deviation for each dilution step, as described above. The LOD and the LOQ were calculated by the addition of 3 or 10 times the standard deviation of the blank sample. For this setup, a LOD of about 1 pM was achieved for Dy-654-COOH. The dynamic range was about a factor of 250, ranging from at least 4 to 1000 pM. A highly linear response (Figure 6, bottom) was achieved for Dy-654-COOH.

### 3.6. Performance of Affinity Column

To evaluate the performance of the TNP-BSA affinity column, a 1:100,000 dilution of the labeled antibody (≈3.5 µg/L) was injected onto the affinity column at varying flow rates, and the signal intensity of the eluate was monitored. At the lowest flow rate of 0.0625 mL min ^−1^ the highest antibody retention of approximately 70% was observed. The antibody removal efficiency gradually declined until the highest flow rate of 0.5 mL min ^−1^ was applied with approximately 54% retention (see Figure S12). When the dead volume of ca. 1 mL is considered (tubing, connectors, and the affinity column), a flow rate of 0.0625 mL min ^−1^ would result in a dead time between injection and measurement of about 16 min. But this delay can be reduced to about 2 min if the highest flow rate of 0.5 mL min ^−1^ would be applied. For all further measurements, a flow rate of 0.5 mL min ^−1^ was used.

### 3.7. TNT Measurements

**Figure 7:**
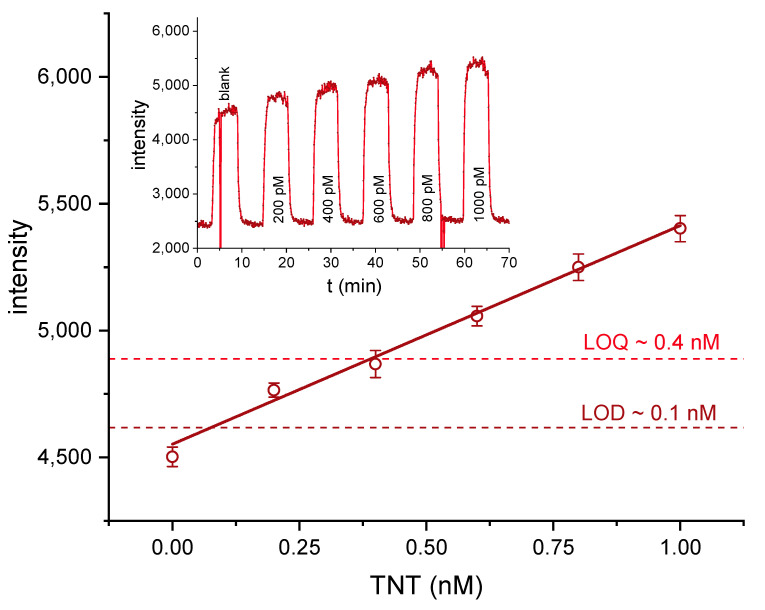
Biosensor measurements of TNT solutions in the range from 0 to 1 nM with a linear fit. LOD (3s) and LOQ (10s) were determined as 0.1 nM (20 ppt) and 0.4 nM (90 ppt). Error bars refer to the standard deviation based on subsequent frames. The biosensor signal is shown above.

Samples from 2 to 10 nM of TNT in 3.5 µg/L of antibody conjugate were incubated for five minutes and injected at 0.5 mL min ^−1^ to determine the dynamic range for the analyte TNT. The results showed that the linear range does not extend well beyond 2 nM for the chosen parameters (see Figure S13). The signal displays an asymptotic behavior. In order to determine the LOD (3s) and LOQ (10s) of the biosensor, dilutions containing 0 to 1.0 nM TNT in 3.5 µg/L labeled A1.1.1 were incubated for five minutes and injected. The raw data were evaluated as described above. From 0 to 1.0 nM TNT, a linear response was observed, and the LOD and LOQ were determined to be about 0.1 nM or 20 ppt TNT and 0.4 nM or 90 ppt TNT, respectively (see Figure 7). 

**Figure 8:**
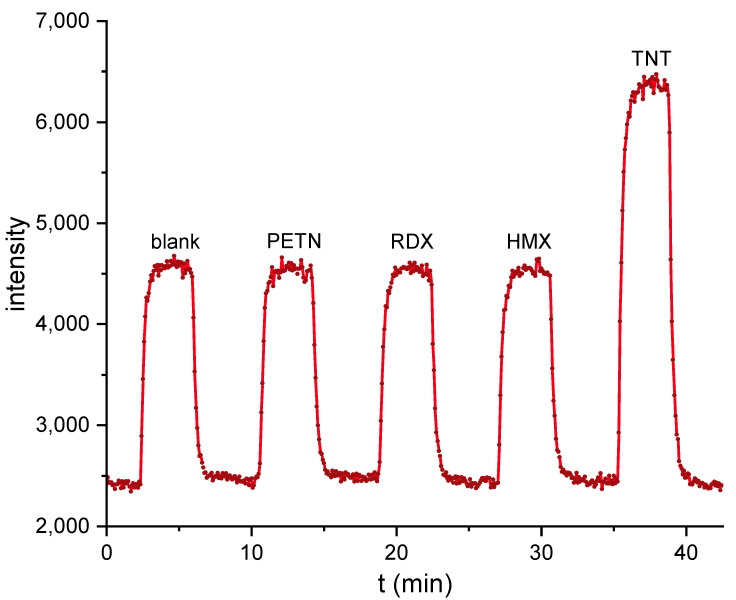
Biosensor-based cross-reactivity test for the high explosives pentaerythritol tetranitrate (PETN), 1,3,5-trinitroperhydro-1,3,5-triazine (RDX), octahydro-1,3,5,7-tetranitro-1,3,5,7-tetrazocine (HMX), and TNT at 5 nM. Only 2,4,6-trinitrotoluene (TNT) shows a positive signal.

Aqueous solutions of the conventional high explosives PETN, RDX, HMX, and TNT (5 nM) were incubated for five minutes with 3.5 µg/L of the labeled antibody and injected. No cross-reactivity could be observed at 5 nM, for all explosives, except TNT (see Figure 8). The results are in good agreement with the detailed characterization of the clone A1.1.1 (see Figure S17 and Table S2) and the literature.

## 4. Discussion

In this paper, a sensitive and fast biosensor for the detection of 2,4,6-trinitrotoluene (TNT) at the ppt (ng/L or pM) level in water is presented. It is based on kinetic competition and hence displays some extraordinary properties. First of all, the biosensor is faster than most competitive immunoassays, which rely on the convergence to a solid-phase equilibrium. In our format, the analyte is incubated in a homogeneous solution with the respective antibody, which is a fast process. In addition, it is advantageous that this biosensor can be considered as quasi-continuous due to the very high capacity of the trapping column. This long-term measurement capability can even be extended by the regeneration of the trapping column, which, however, is not shown here. Another advantage is the calibration curve, which displays a positive slope in contrast to conventional competitive assays. There is a small delay of about two minutes between the analyte injection and the signal increase due to the dead volumes in the system. Shorter connections and higher flow rates could reduce this in the future.

Although the general setup of the system consisting of a wide-field epifluorescence system is quite common, one of the aims of the project was to establish a highly sensitive laser-induced fluorescence detector (LIF), with low-cost equipment (see Figure S4). For many applications, the budget is limited, and hence, systems based on expensive high-end components may not find broad application. Fortunately, the prices of many semiconductor devices, such as cooled charge-coupled device (CCD) or CMOS cameras, have dropped considerably, without compromising their performance. 

Another decision is the choice of a suitable (fluorescence) label. During the last decades, many improved fluorescence dyes became commercially available, showing better quantum yield, ozone, bleaching and pH stability, less tendency for aggregation, water-solubility, ease of conjugation, and many more. Finally, we have chosen a highly hydrophilic dye, which can be excited with a wavelength of about 635 nm, for which small and cheap diode lasers are available. Perhaps it should be noted that other similar dyes also might be suitable for this purpose.

One of the most critical and often neglected issues is the selection of the antibody. Only very few (monoclonal) antibodies against explosives or TNT, respectively, are available. We were able to purchase two monoclonal anti-TNT-antibodies, the clones A1.1.1 and EW75C. We have characterized both antibodies in detail to determine their sensitivity and their cross-reactivity patterns. In most cases, the cross-reactivities (CR) were relatively similar (see Table S2). Both clones displayed a high cross-reactivity against 2,4,6-trinitroaniline and a relatively high CR against 2,4,6-trinitrobenzene and some dinitrobenzenes or dinitrotoluenes. A significant difference is the lack of CR against any nitro musk compounds of the clone EW75C, in contrast to A1.1.1 (see Table S2). The opposite is the case with some nitrophenyl alkyl acids. The most relevant difference, however, is the test midpoint in ELISA format, which is around 1.2 nM (270 ng/L) for A1.1.1 and 36 nM (8.2 µg/L) for EW75C (see Figure S17). Due to this significant sensitivity difference, we have chosen to proceed with A1.1.1 only. For identification purposes for both clones, antibody fingerprints have been prepared, which are shown in the Supplementary Materials (Figure S19).

Liquid handling is another issue in biosensor development. In our system, we rely on high-performance syringe pumps, which are robust, not too expensive, and deliver a nearly pulsation-free flow. The sample and the antibody are premixed and injected with a conventional 6-port injection valve. A real sampling system for air or wipe tests is lacking, however. The antibody trap is based on a monolithic affinity column developed in-house. It is manufactured from partially sintered borosilicate glass powder and coated via silane chemistry and conjugation with trinitrophenyl-derivatized albumin. The glass monolith is glued into a titanium shell and attached to standard high-performance liquid chromatography (HPLC) fittings. These glass monoliths have the advantage that they tolerate high flow rates while displaying low backpressures and show fast binding kinetics due to a lack of internal porosity. As a miniaturized flow-cell, a microfluidic COC chip was used. This polymer shows excellent optical properties, which are similar to glass, and is resistant to most chemical attacks, as from acids and bases.

The optical system consists of a Zeiss objective for microscopes, a dichroic mirror, different filters, and a diode laser (635 nm, <1 mW). A cooled CMOS camera containing a Sony IMX-174M chip was used as the optical detector. In total, the cost of this setup was about <5000 EUR (including taxes, S4), which is quite moderate for this level of performance.

In order to characterize the system, several tests have been performed. First of all, the sensitivity of the detector was examined with dilutions of the fluorescence dye (Dy-654-COOH). A highly linear relationship was obtained in the concentration range of 0–1000 pM. A limit of detection (LOD) of about 1 pM was found (Figure 6). In another experiment, the trapping efficiency of the affinity column was examined (Figure S12). An efficiency of 60–70% was achieved, which might indicate some impurities of the antibody. Any inactive antibody will lead to a higher background signal. Due to the shorter response time, the faster flow rate of 0.5 mL/min was chosen. In Figure 7, the detection of TNT was tested with the setup shown in Figure 2 and Figure 3. A LOD of approximately 0.1 nM or 20 ppt TNT was achieved, which is significantly lower than the LOD of 60 ppt of a highly optimized competitive ELISA [45], and surpassing most biosensors based on the same antibody [4,26,30,31,38,71,72,73,74,75,76,77,78,79,80,81,82,83,84].

Finally, some basic cross-reactivity tests with high explosives have been performed with the new biosensor format. It could be shown that only TNT leads to a significant signal at 5 nM. PETN, RDX, and HMX did not show any increased signal, even at much higher concentrations.

## 5. Conclusions

It could be shown that biosensors based on kinetic competition are very powerful and promising systems for the fast and highly sensitive detection of explosives, such as TNT. In contrast to many other biosensors presented for TNT and other nitroaromatic compounds, this approach does not depend on any specific physicochemical property of the target compound. Therefore, it can be easily transferred to all other explosives and substances of interest, for which suitable monoclonal antibodies or similar binders are available today or can be made. It has to be mentioned that monovalent binders, such as Fab fragments, are preferable to bivalent antibodies in this assay format to achieve maximum sensitivity. High speed and excellent sensitivity are also striking advantages. Although these systems are highly specific and hence primarily designed for mono-analyte detection, the setup can be easily parallelized and, therefore, transformed into a multiplex biosensor system. In particular, the use of a conventional CMOS camera offers the opportunity to detect many signals in parallel without the need for an additional detector or the use of highly expensive EMCCD cameras. Similarly, the beam of the laser diode might be split into several sub-beams to excite several flow channels on one chip at a time. The running costs of such a biosensor would be lower, as many would expect from an immunochemical device. With only one milligram of antibody, the biosensor could be operated continuously for more than one year. Finally, it has to be stressed that due to the use of extraordinarily selective antibodies as binders, these biosensors are not prone to false-positive signals, which is crucial for any real-world application in the security field.

## Supplementary Materials

The following are available online at https://www.mdpi.com/2079-6374/10/8/89/s1: Preparation of the affinity column, Optical setup of the fluorescence detector, Sensor optimization, Mass spectrometric conjugate characterization, calibration curves and precision profiles of the competitive indirect ELISAs based on the TNT antibodies EW75C and A1.1.1; Determination of cross-reactivities, MALDI-TOF MS antibody fingerprints of the monoclonal antibodies A1.1.1 and EW75C, Description of the semi-automated data evaluation with the python script, Determination of the coordinates of the laser point.
