# Supplementary material for: Fast Detection of 2,4,6-Trinitrotoluene (TNT) at ppt Level by a Laser-Induced Immunofluorometric Biosensor
Source: Biosensors (Basel). 2020 Aug 5;10(8):89. doi: 10.3390/bios10080089 (PMC7460505; doi:10.3390/bios10080089)
Supplement: Supplementary file 1 [file biosensors-10-00089-s001.zip › Paul-Biosensors-Supplementary-Materials.pdf]

biosensors

MDPI

Supplementary Materials

Fast Detection of 2,4,6-Trinitrotoluene (TNT) at ppt Level by a Laser-Induced Immunofluorometric Biosensor

Martin Paul [ORCID], Georg Tscheuschner [ORCID], Stefan Herrmann [ORCID], Michael G. Weller [ORCID]*

Federal Institute for Materials Research and Testing[1] (BAM), Division 1.5 Protein Analysis, Richard-Willstätter-Strasse 11, 12489 Berlin, Germany,
* Correspondence: michael.weller@bam.de; Tel.: +49-30-8104-1150

Preparation of the affinity column

For the column functionalization, the raw columns were treated as described in *Table S1*. The silanization solution contained 1% (v/v) of lab water and 1% (v/v) of diethoxy(3-glycidyloxypropyl)-methylsilane (97%) in absolute ethanol. After the affinity coating, the column was stored under 80 % ethanol at 4°C and remained stable for several months.

Table S1. Preparation of the trinitrophenyl-(TNP)-BSA column

**Reagent**	**Time (min)**	**Flow rate (mL min^{-1})**	**Volume (mL)**
Water	5	1	5
KOH 10 %	9	0.5	4.5
Water	5	1	5
HCl 1 M	60	0.1	6
PBS	5	1	5
Water	5	1	5
EtOH	20	1	20
Silanization solution	3	1	3
Storage at RT	28 h	-	
EtOH	20	1	20
PBS	10	1	10
TNP-BSA	2.5	1	2.5
Incubation, RT	144 hours	-	-
PBS	20	1	1
EtOH/Water 80:20	10	1	1

[1] Bundesanstalt für Materialforschung und -prüfung (BAM), www.bam.de

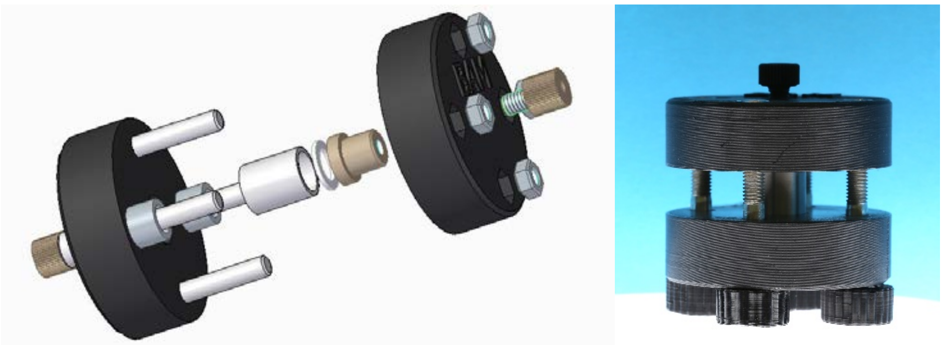

Figure S1. Engineering drawing of the column holder made by additive manufacturing (Solid Edge; Bettina Roeder, BAM) with custom 1/16" PEEK fittings and monolithic affinity column (left), and photo (Martin Paul, BAM) operational column ready to use (right).

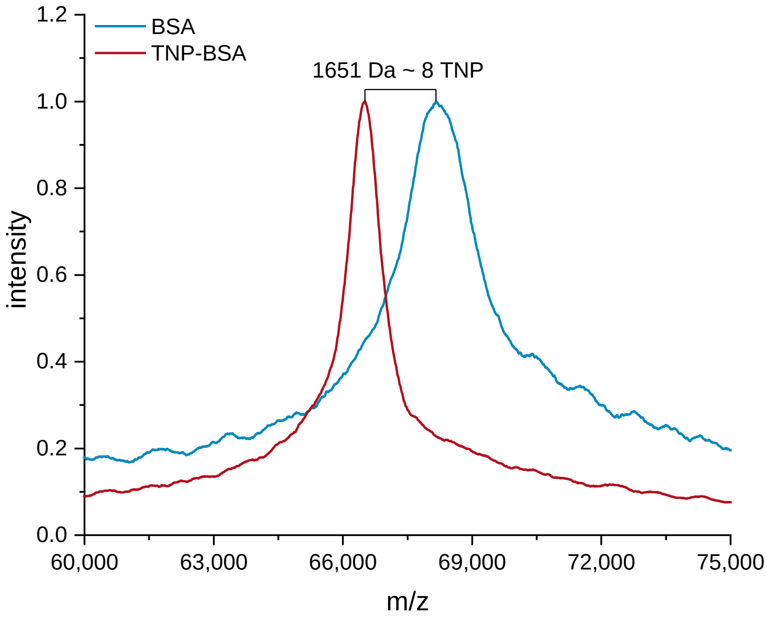

Figure S2. Smoothed MALDI-TOF MS of the TNP-BSA conjugate for the coating of the affinity column with a degree of labeling (DOL) of approx. 8 trinitrophenyl groups per protein.

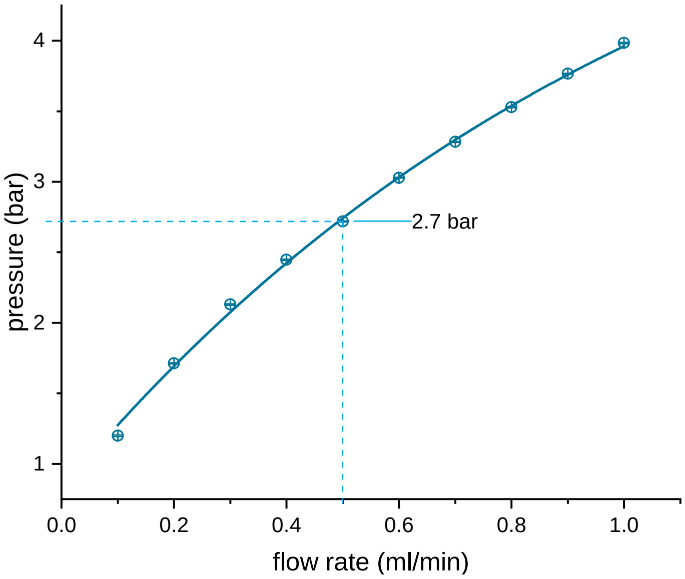

Figure S3. Backpressure of a Vitrapor5 column with water; the value for the usual flow rate of 0.5 mL min^{-1} is indicated. Measurement performed by Marco Wilke, BAM.

Optical setup of the fluorescence detector

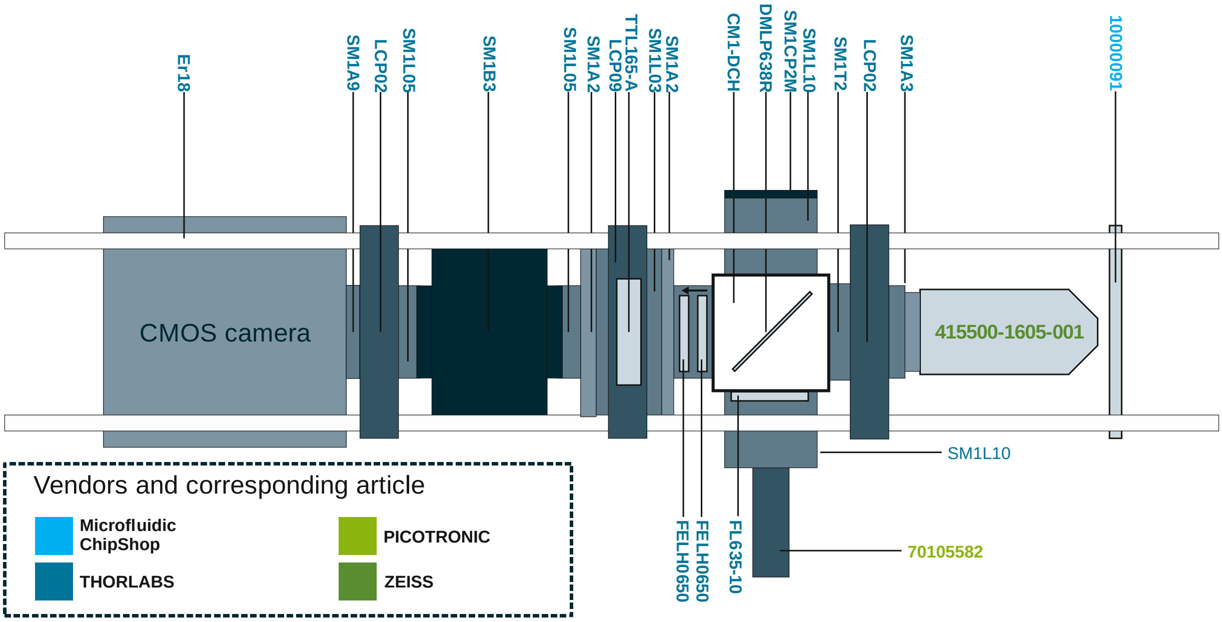

Figure S4. Optical setup of the detector, including articles color-coded by their vendors. The CMOS camera was a QHY174M GPS system. The shown parts may be acquired for < 5000 € (incl. taxes) as 06/2020.

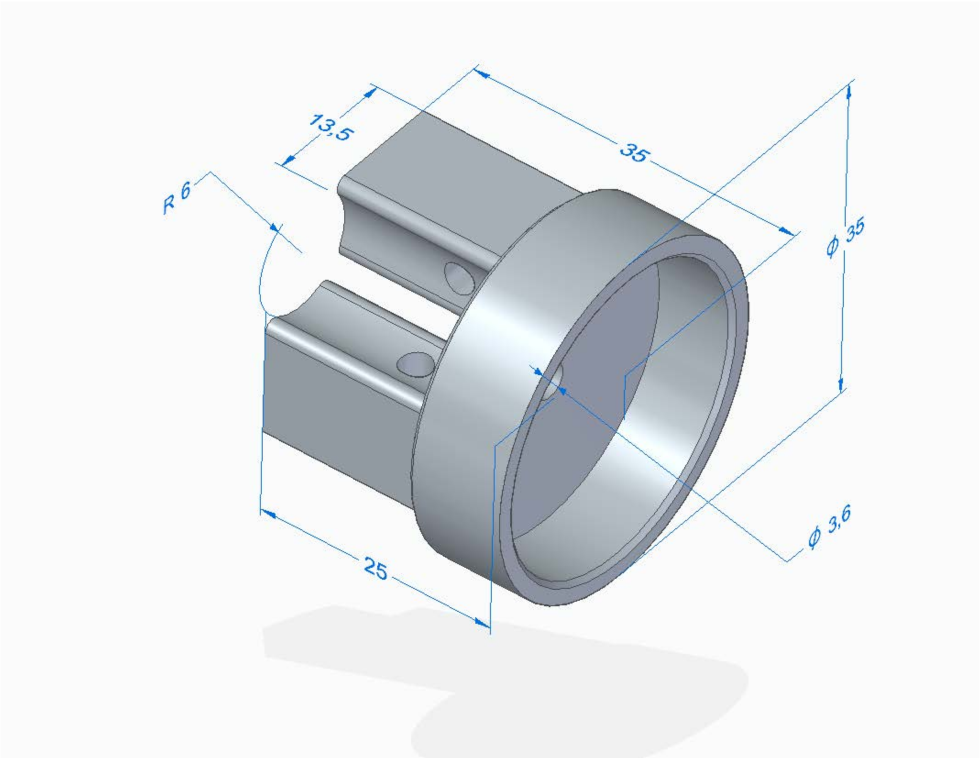

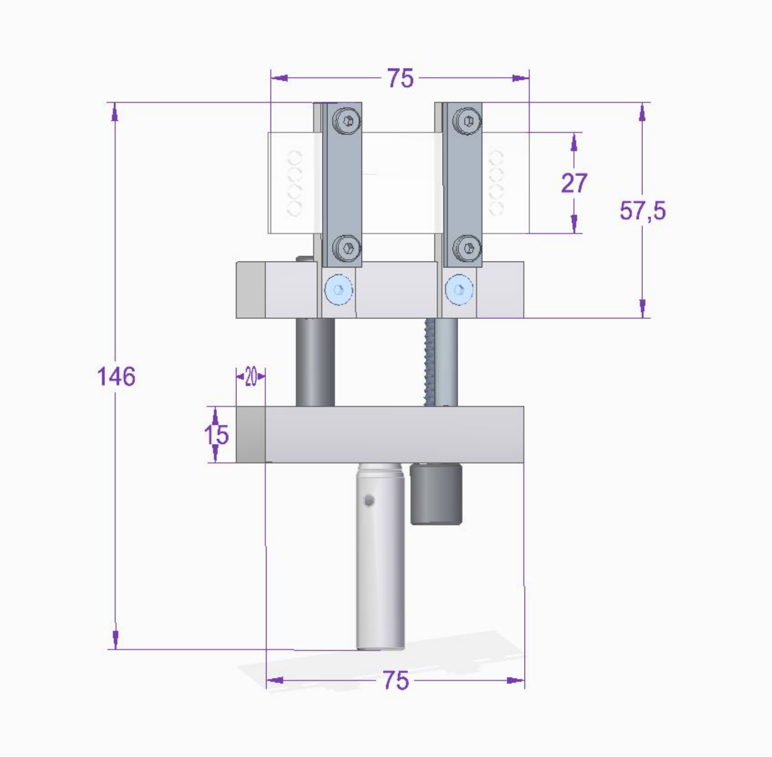

Figure S5. Engineering drawing of the laser holder made by additive manufacturing (Solid Edge, Bettina Roeder, BAM), which allows for convenient adjustment of the laser spot (top) and the holder for the microfluidic chip (bottom), which enables the height-adjustment of the chip (Solid Edge; Bettina Roeder, BAM). All given dimensions are stated in mm.

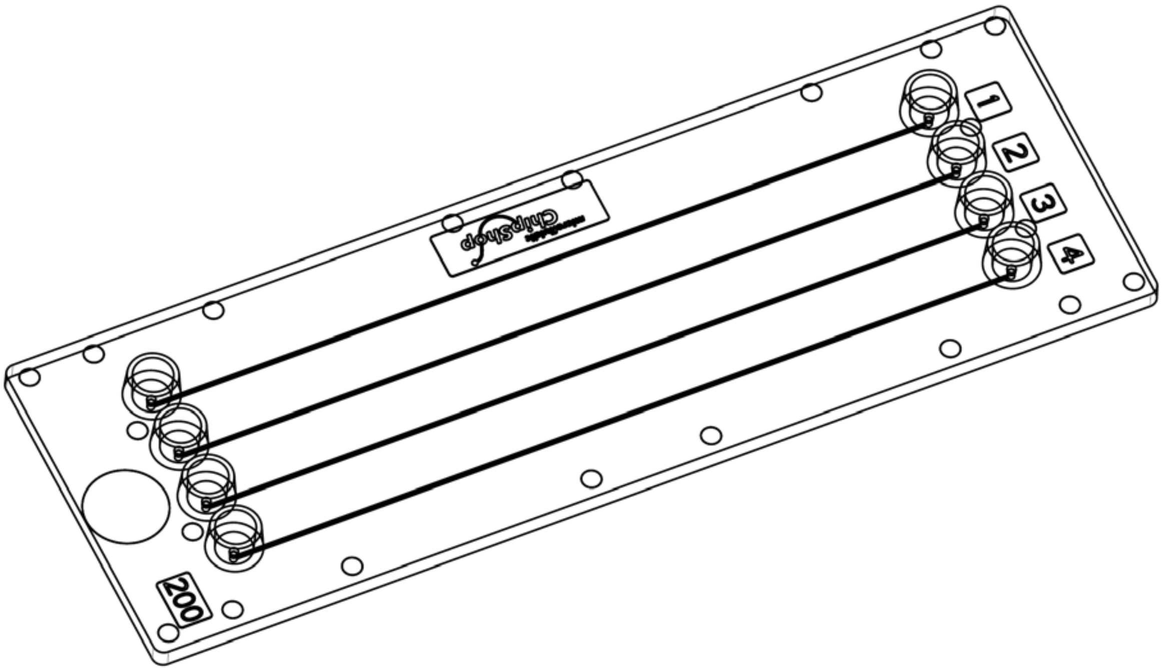

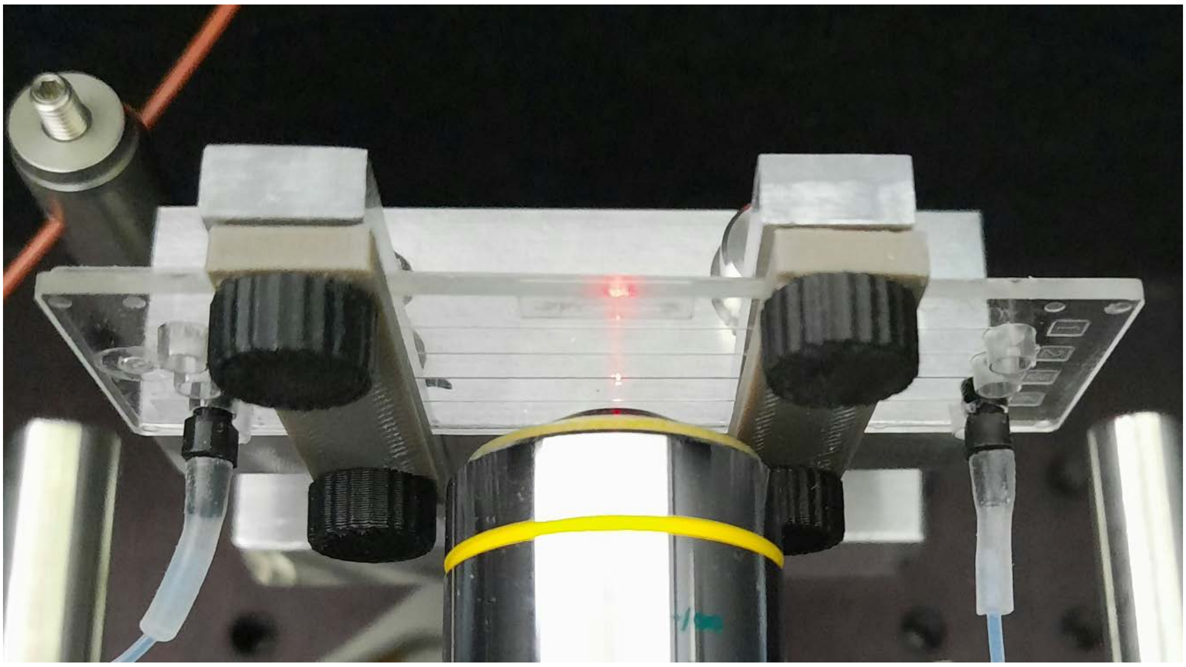

Figure S6. Straight channel chip (10000091, microfluidic ChipShop), the layout was provided by microfluidic ChipShop, Jena, and photo of the installed chip with active laser (bottom) by Martin Paul, BAM.

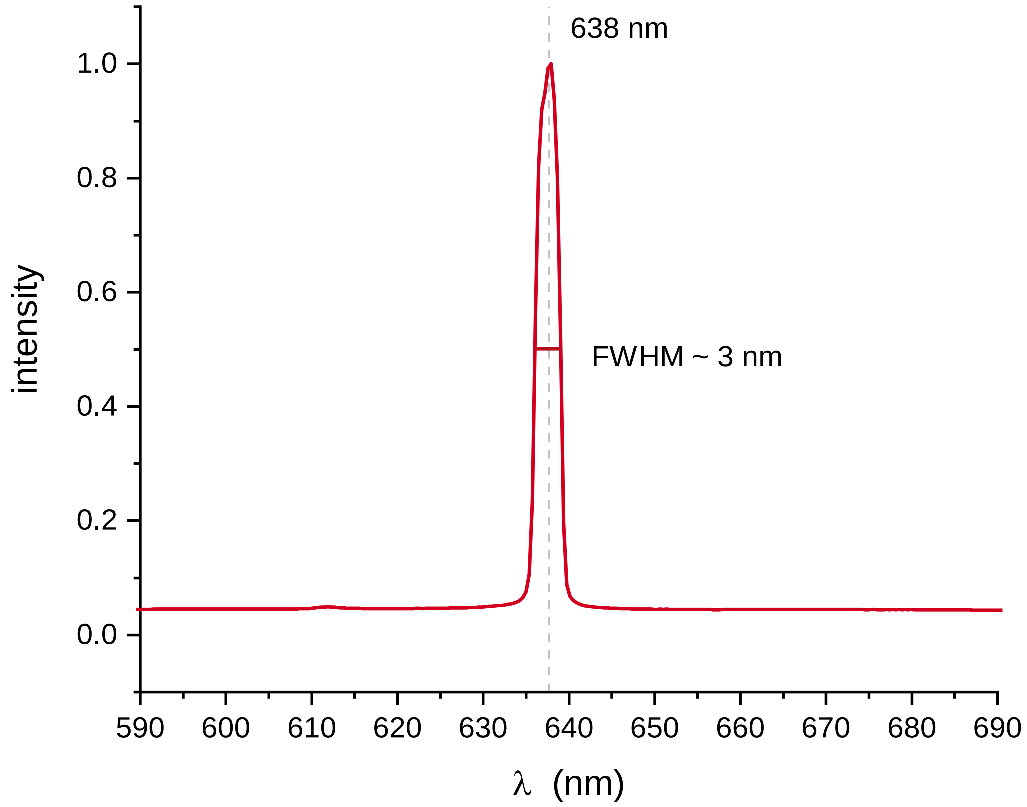

Figure S7. The emission spectrum of the laser diode (70105582, Picotronic) was determined to have a center wavelength of 638 nm and a full width at half maximum (FWHM) of approx. 3 nm. The measurement was performed by Florian Weigert, BAM.

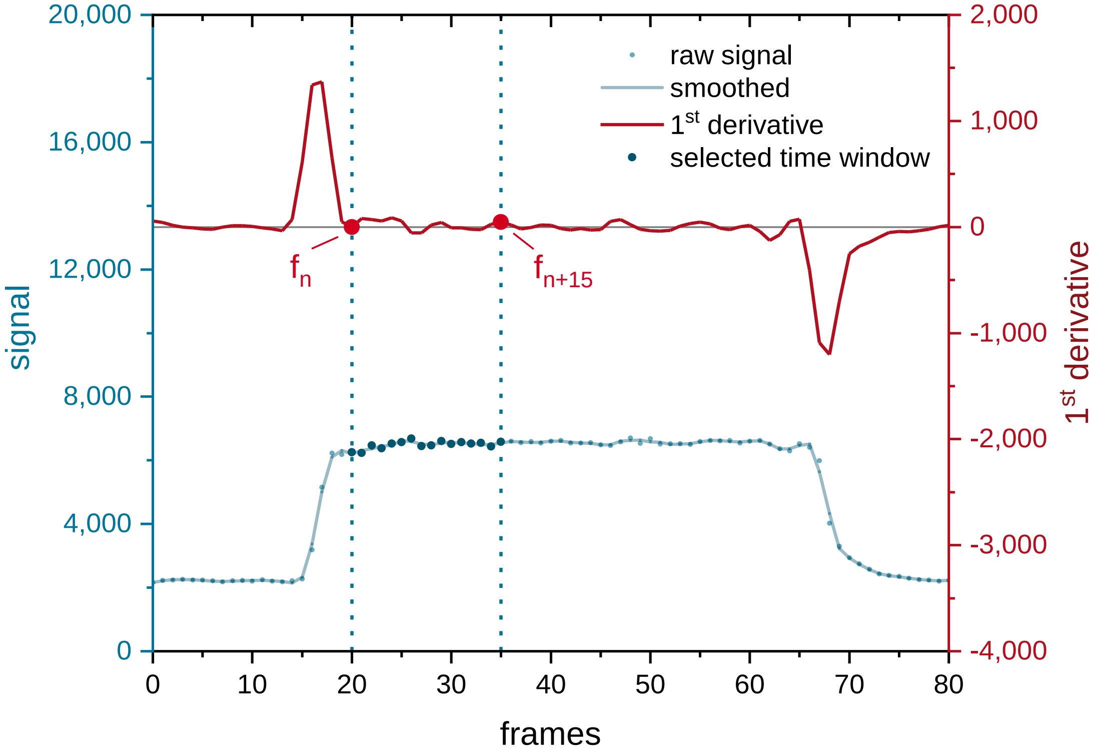

Figure S8. Determination of the first frame to be evaluated (f_n) based on the 1st derivative of the Savitzky–Golay-smoothed data shown for a sample injection of 100 pM Dy-654-COOH in PBS.

Sensor optimization

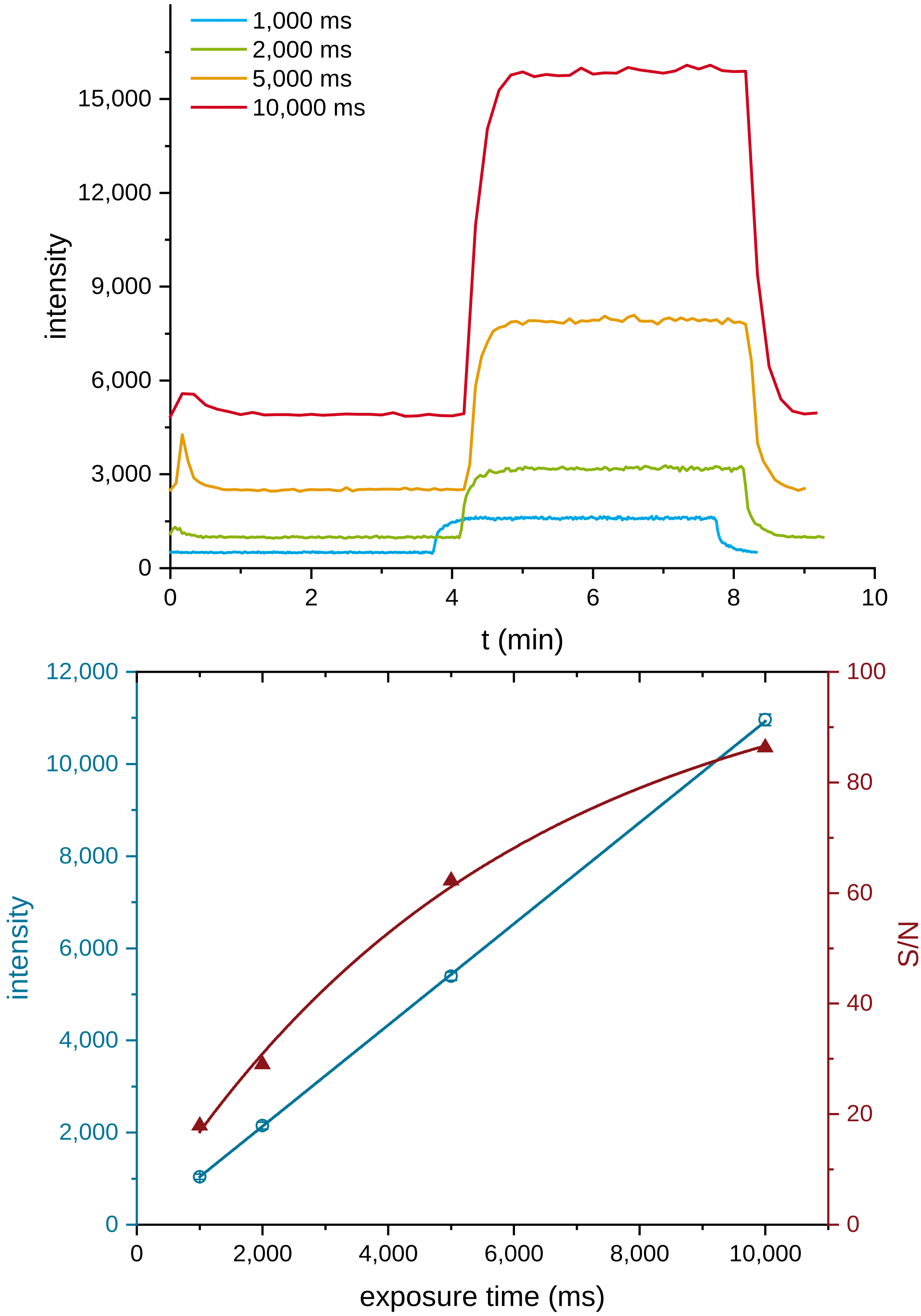

Figure S9. Comparison of the sensor performance at a sensor temperature of -5°C and a gain of four with exposure times ranging from 1,000 to 10,000 ms based on measurements of PBS from 0 to 3 minutes and 100 pM Dy-654-COOH from 4 to 7 minutes (top). For each measurement, the mean and standard deviation of 16 frames of the blank and the label were determined. The intensity difference between the label and the blank was divided by the sum of the standard deviation of the blank and the label and plotted as S/N (bottom).

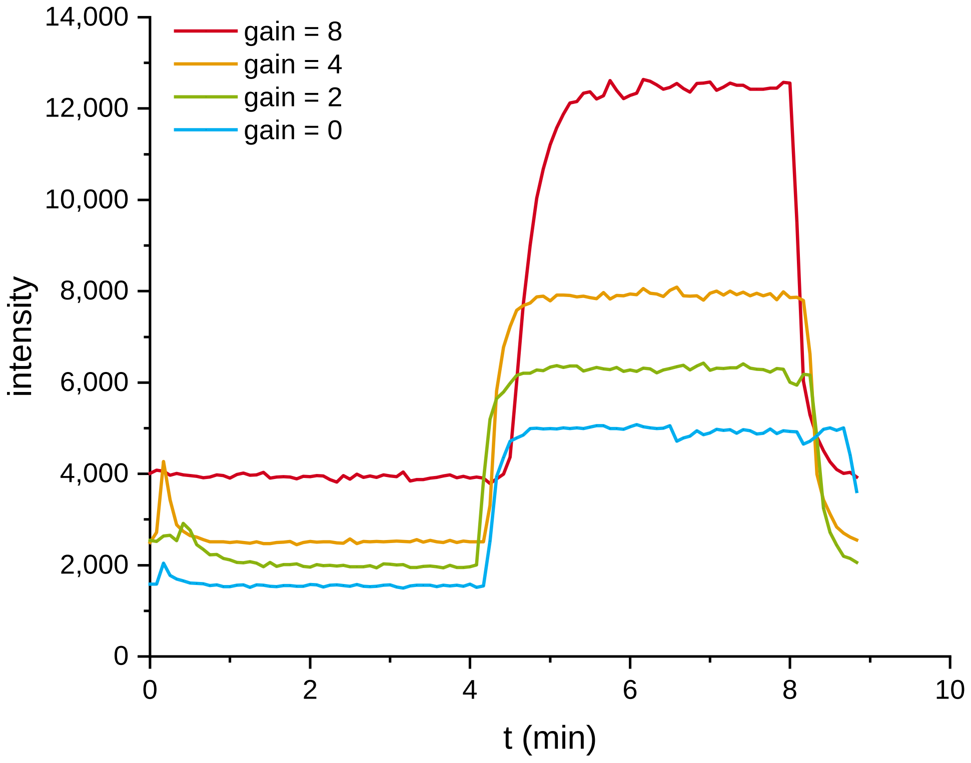

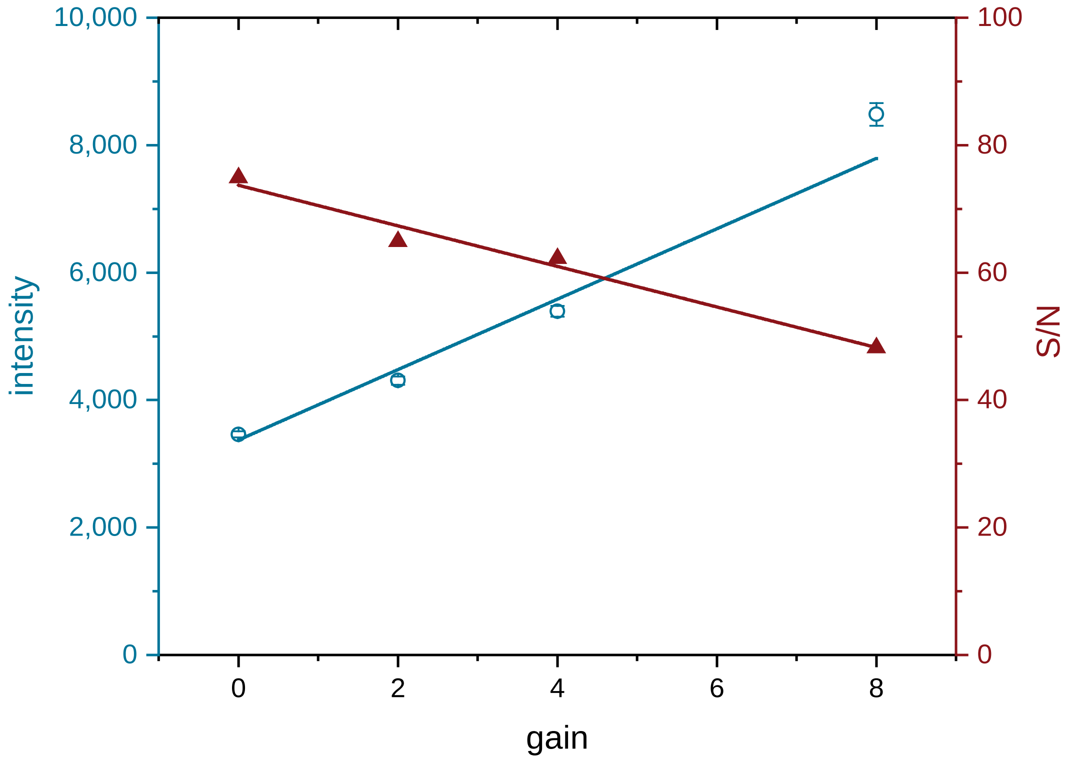

Figure S10. Comparison of the sensor performance at a sensor temperature of – 5°C and exposure time of 5000 ms and gain ranging from 0 to 8 based on measurements of PBS from 0 to 3 minutes and 100 pM Dy-654-COOH from 4 to 7 minutes (top). For each measurement, the mean and standard deviation of 16 frames of the blank and the label were determined. The intensity difference between the label and the blank was divided by the sum of the standard deviation of the blank and the label and plotted as S/N (bottom).

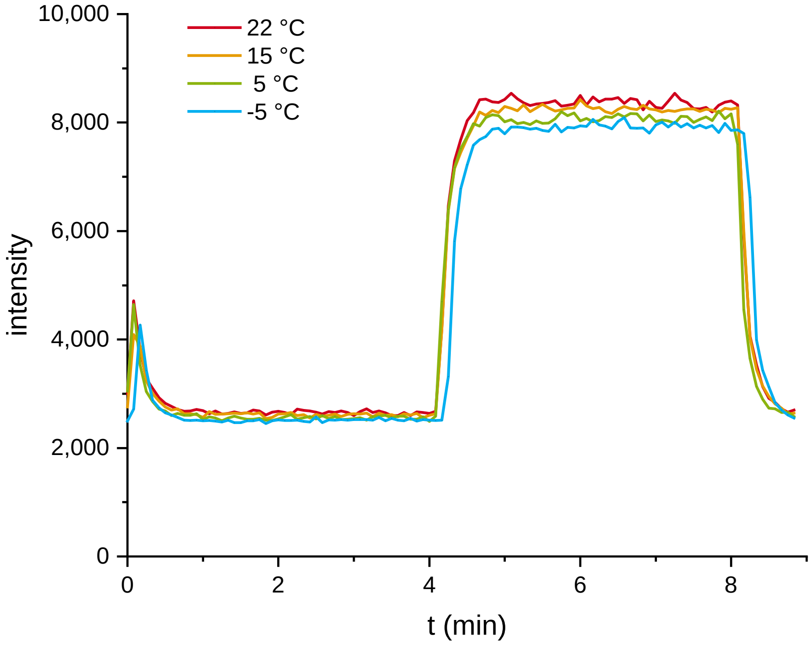

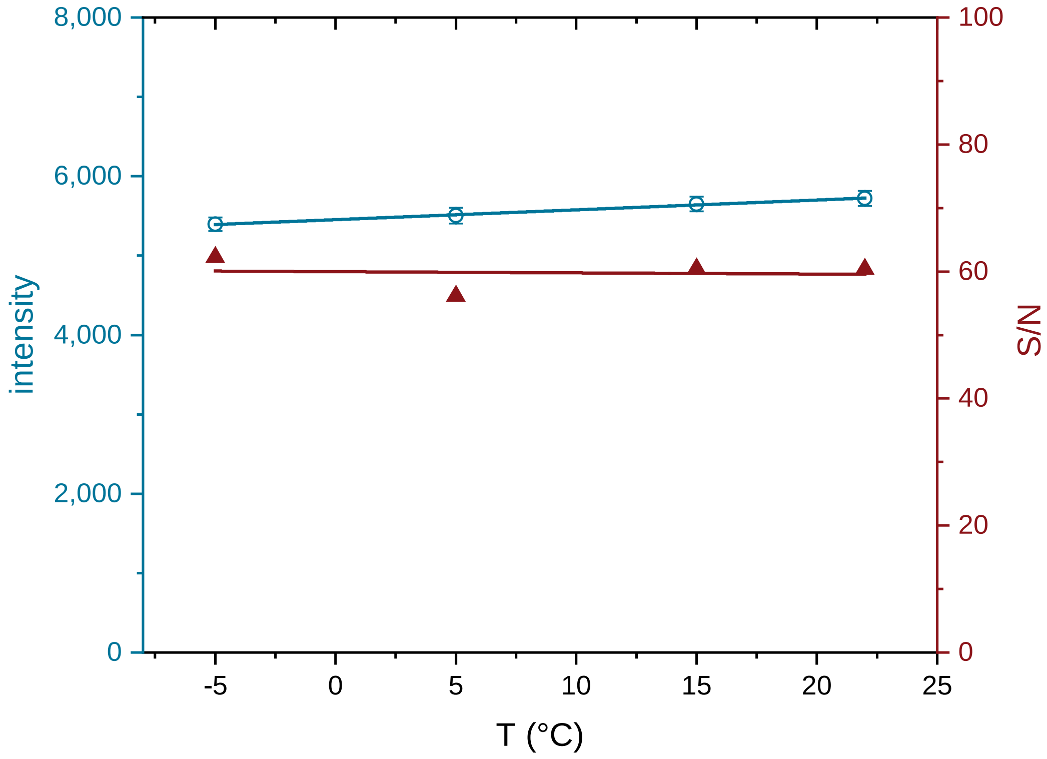

Figure S11. Comparison of the sensor performance at varying sensor temperatures ranging from -5 °C to 23 °C based on measurements of PBS from 0 to 3 minutes and 100 pM Dy-654-COOH from 4 to 7 minutes (top). For each measurement, the mean and standard deviation of 16 frames of the blank and the label were determined. The intensity difference between the label and the blank divided by the sum of the standard deviation of the blank and the label is plotted as S/N (bottom).

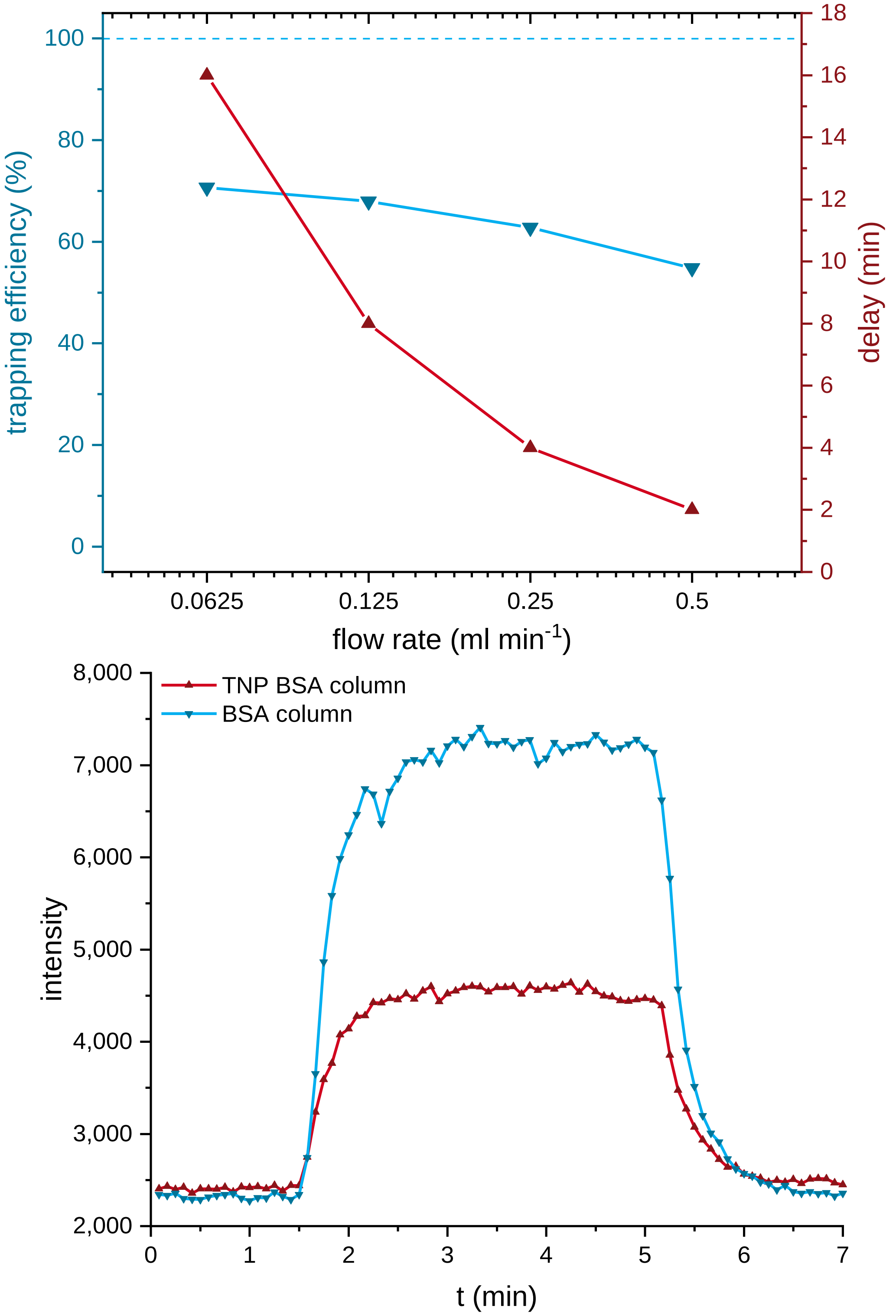

Figure S12. Influence of the flow rate on the trapping efficiency of the labeled antibody and the delay time (top). Comparison of the antibody retention of the TNP-BSA affinity column (red) and the BSA column (blue) for 3.5 µg/L of antibody conjugate at a flow rate of 0.5 mL min^{-1} (bottom).

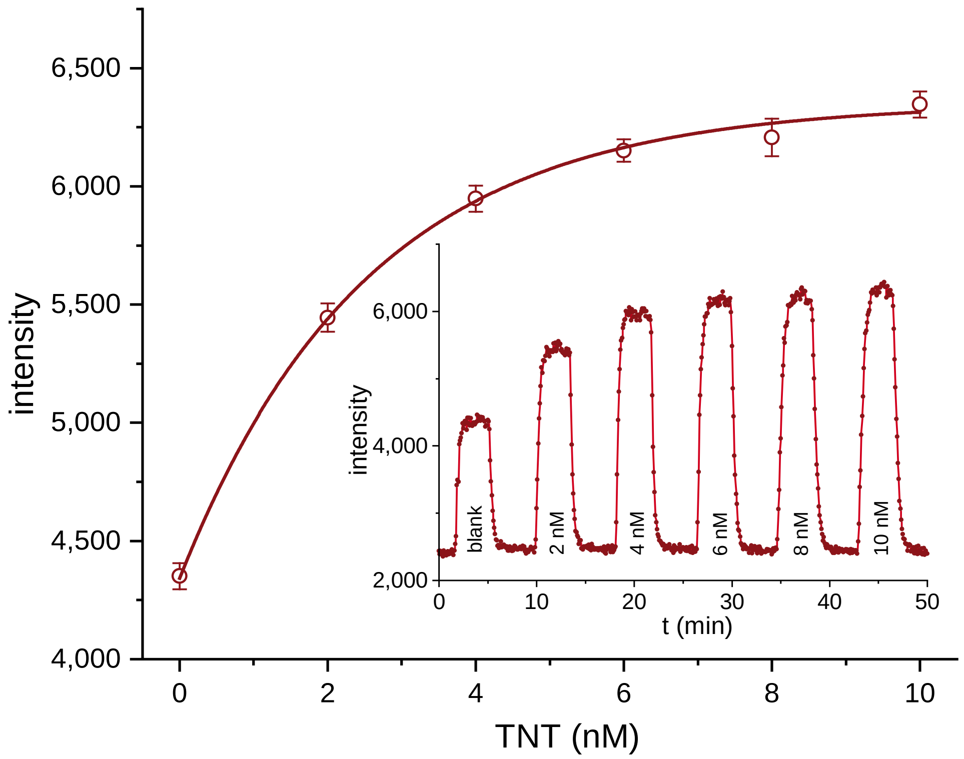

Figure S13. Detection range for TNT (2-10 nM, high range) as raw data (insert) and evaluated with an asymptotic fit.

Mass spectrometric conjugate characterization, calibration curves and precision profiles of the competitive indirect ELISAs based on the TNT antibodies EW75C and A1.1.1; determination of cross-reactivities

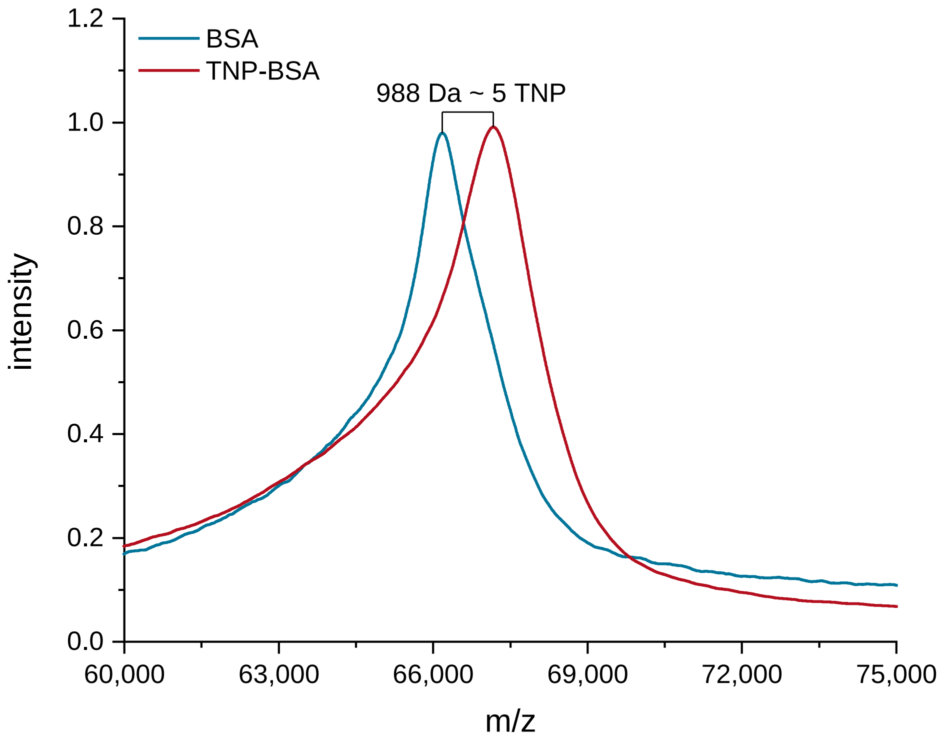

Figure S14. Smoothed MALDI-TOF MS of the TNP-BSA conjugate for the competitive ELISA with a degree of labeling (DOL) of approx. 5.

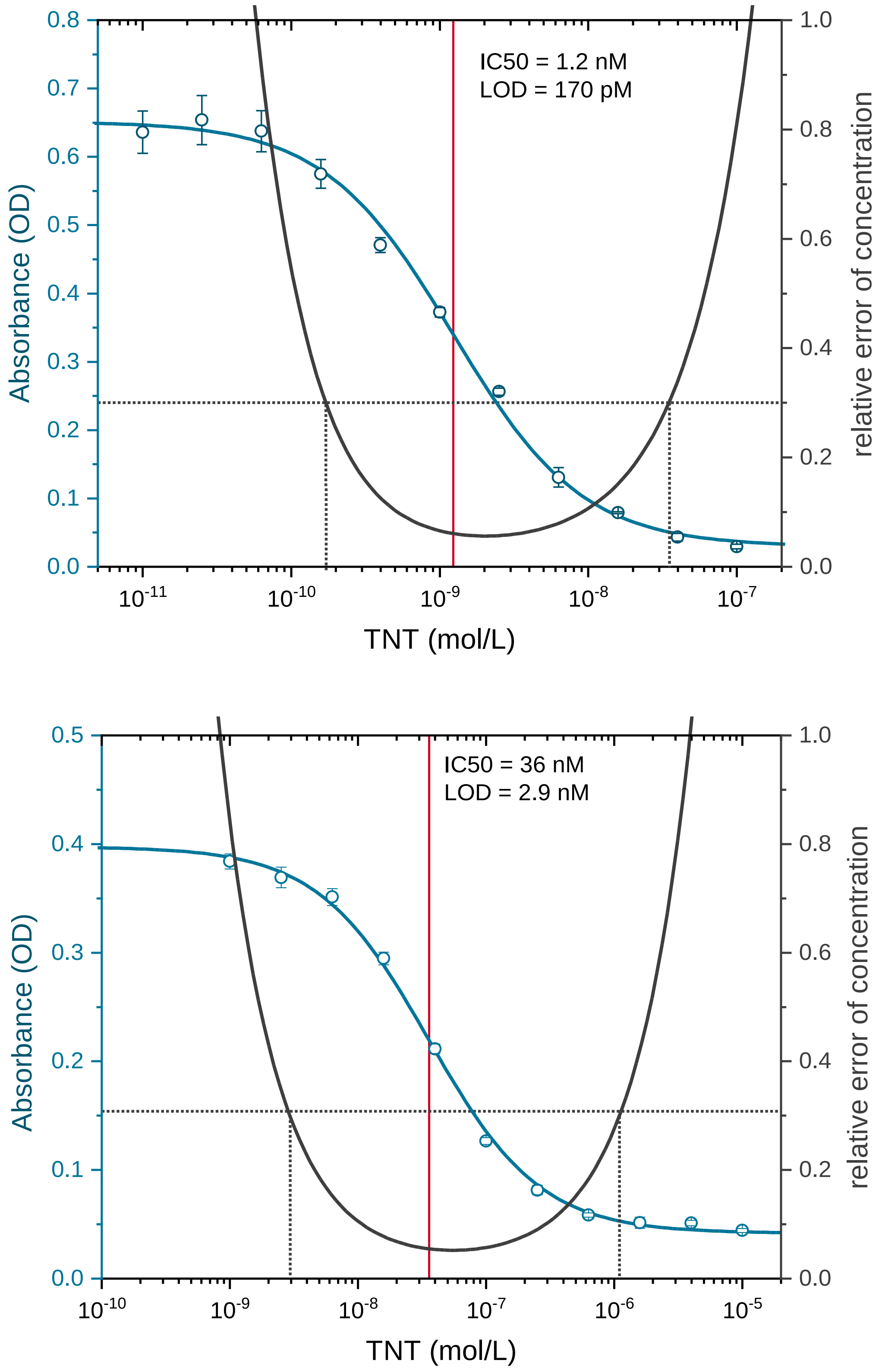

Figure S15. Calibration curves and precision profiles of the clone ***A1.1.1 (top)****, and the clone* ***EW75C (bottom)*** *with the analyte TNT as quadruplicates. The clone A1.1.1 shows superior performance compared to EW75C.*

In order to obtain the relative error of a concentration determination (Δx), a three parameters logistic regression (see Formula S1) of the ELISA signal is used to determine the slope of the curve as the first derivative (Formula S2). The standard deviations of the replicates are fitted with a four-parameter logistic regression (see Formula S1). Both functions are combined to calculate the relative error (Δx) (Formula S3).

$$f(x) = A2 + \frac{(A1 - A2)}{\left(1 + \left(\frac{x}{x0}\right)^{p}\right)}$$

Formula S1: Four-parameter logistic regression; for the three-parameter logistic regression, p = 1. Here, A2 and A1 are the lower and upper asymptotes, and x0 is the test midpoint of the assay.

$$\frac{df(x)}{dx} = \frac{-(A1 - A2)}{x0 \cdot \left(1 + \frac{x}{x0}\right)^{2}}$$

Formula S2: First derivative for the three parameters logistic regression.

$$\Delta x = -\frac{std(x)}{x \cdot \frac{df(x)}{dx}}$$

Formula S3: Calculation of the relative error with the fitted standard deviation (std(x)) and the slope of the ELISA signal.

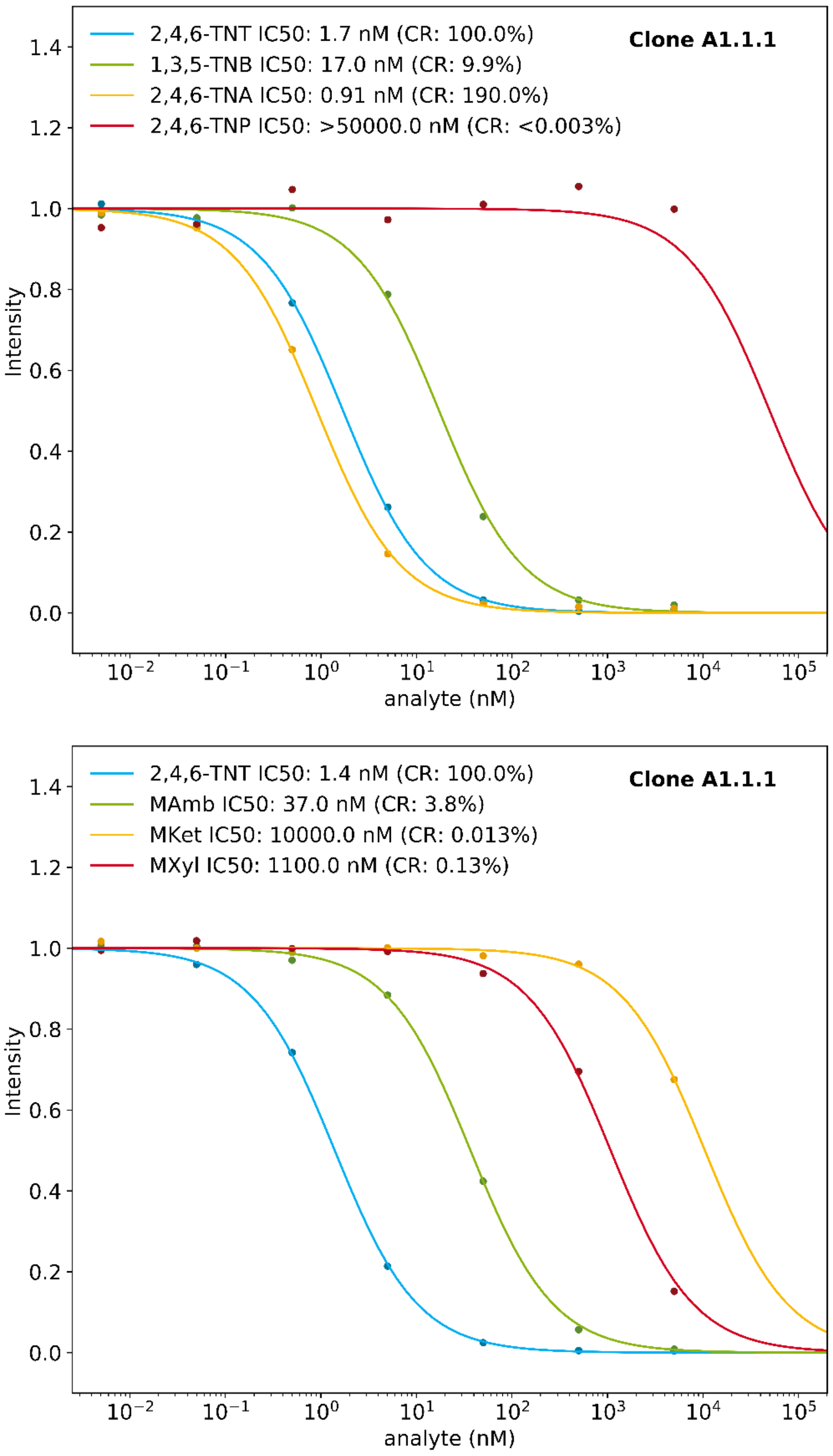

Figure S16. Three-parameter, logistic fitted, normalized, background-subtracted cross-reactivities of the clone A1.1.1 for aromatic trinitro derivates: 2,4,6-trinitrotoluene (2,4,6-TNT), 1,3,5-trinitrobenzene (1,3,5-TNB), 2,4,6-trinitroaniline (2,4,6-TNA) and 2,4,6-trinitrophenol (2,4,6-TNP; picric acid) (top) and important nitro musks (fragrances): musk ambrette (MAmb), musk ketone (MKet) and musk xylene (MXyl).

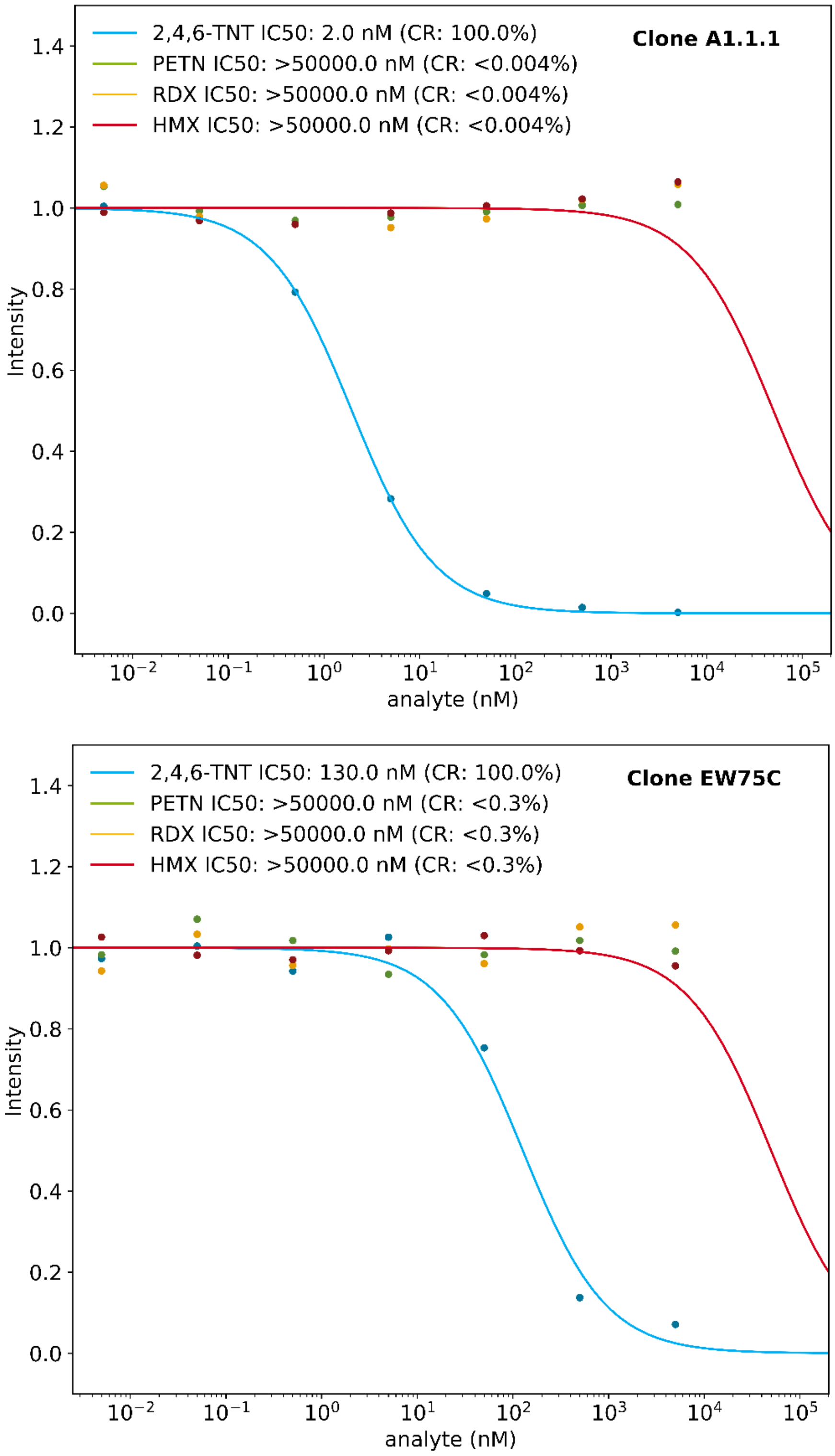

Figure S17. Three-parameter logistic fitted, normalized, background-subtracted cross-reactivities for the high explosives TNT, PETN, RDX, and HMX for the clones A1.1.1 (top) and the clone EW75C (bottom). While both clones show no cross-reactivity for the high explosives, the clone A1.1.1 has a significantly lower test midpoint.

Table S2. Cross-reactivities (CR) relative to TNT of the clones A1.1.1 and EW75C in % based on competitive indirect ELISA (antigen-immobilized).

Substance	Abbreviation	A1.1.1 (CR %)	EW75C (CR %)
2,4,6-Trinitrotoluene	2,4,6 TNT	100	100
2-Nitrotoluene	2NT	<0.005	<0.3
3-Nitrotoluene	3NT	<0.005	<0.3
4-Nitrotoluene	4NT	<0.005	<0.3
2,3-Dinitrotoluene	2,3DNT	0.046	1.8
2,4-Dinitrotoluene	2,4DNT	1.9	6.9
3,4-Dinitrotoluene	3,4DNT	0.022	<0.3
1,2-Dinitrobenzene	1,2DNB	<0.006	8.3
1,3-Dinitrobenzene	1,3DNB	0.16	7.4
Nitrobenzene	NB	<0.006	<0.6
1,3,5-Trinitrobenzene	1,3,5-TNB	9.9	38
2,4,6-Trinitroaniline	2,4,6-TNA	190	1800
2,4,6-Trinitrophenol	2,4,6-TNP	<0.003	<0.3
2-Methyl-3-Nitroaniline	2-M-3-NA	<0.005	<0.3
2-Methyl-4-Nitroaniline	2-M-4-NA	<0.005	<0.3
2-Methyl-6-Nitroaniline	2-M-6-NA	<0.005	<0.3
1,3-Dinitrophthalate	1,3-DNPth	8.1	8.1
1,4-Dinitrobenzene	1,4-DNB	0.019	<0.3
Nitroguanidine	NGua	<0.004	<0.3
2-Nitrophenylacetic acid	2-NPAAh	0.0074	0.71
3-Nitrophenylacetic acid	3-NPAA	<0.004	0.94
4-Nitrophenylacetic acid	4-NPAA	<0.004	0.67
4-Nitrophenylbutyric acid	4-NPBA	<0.008	37
2,4-Nitrophenylacetic acid	2,4-DNPAA	0.034	2.2
1,8-Dinitronaphthalene	1,8-DNNapth	0.076	1.5
PETN	PETN	<0.004	<0.3
RDX	RDX	<0.004	<0.3
HMX	HMX	<0.004	<0.3
Musk ambrette	MAmb	3.8	<0.3
Musk ketone	MKet	0.013	<0.3
Musk xylene	MXyl	0.13	<0.3

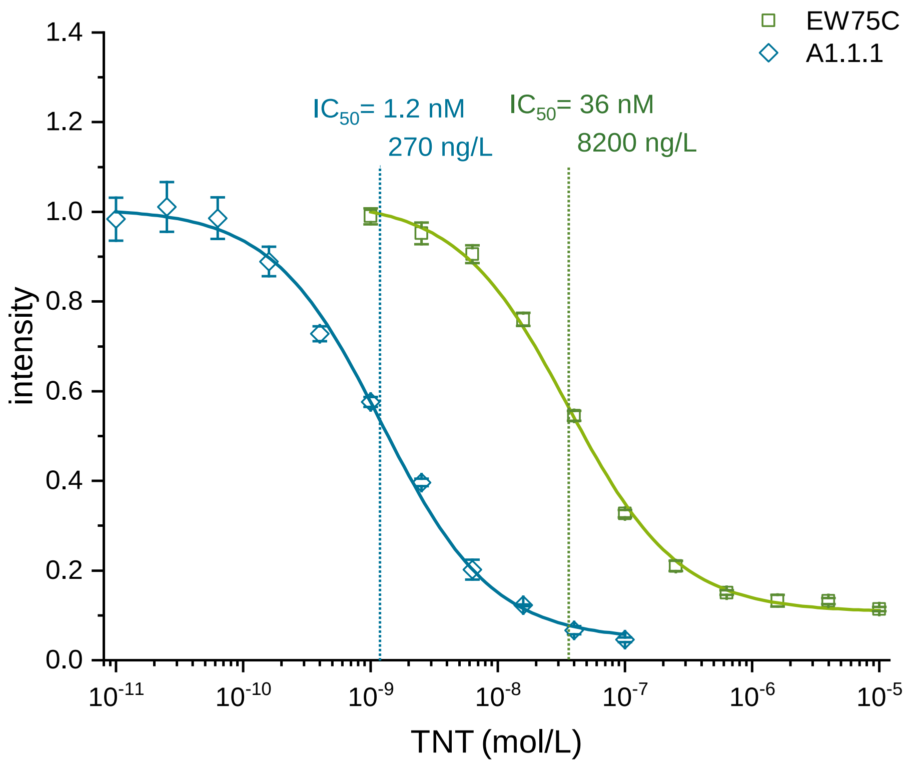

Figure S18 Comparison of the clone A1.1.1 and EW75C in indirect competitive ELISA. The detection limit for the clone A1.1.1 was determined to be 0.17 nM or 39 ng/L, the IC50 at 1.2 nM or 270 ng/L, and the upper limit of quantification was determined to be 35 nM or 8000 ng/L. For the clone EW75C, the detection limit was determined to be 2.9 nM or 0.66 µg/L, the IC50 at 36 nM or 8.2 µg/L, and the upper limit of quantification was determined to be 1100 nM or 250 µg/L.

Antibody fingerprints of the monoclonal antibodies A1.1.1 and EW75C against TNT

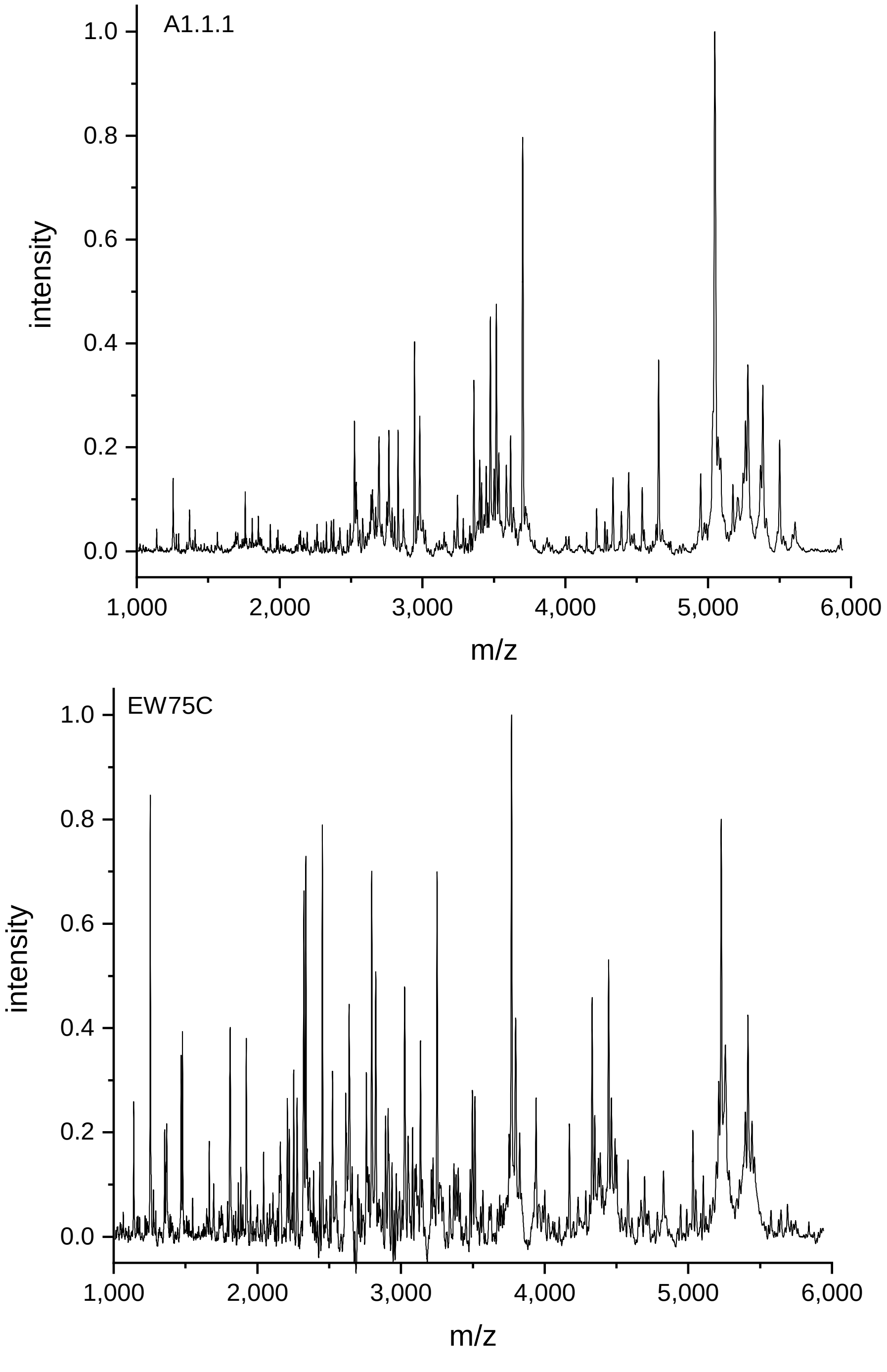

Figure S19: MALDI-TOF MS antibody fingerprints, according to Tscheuschner et al.[2] *of clone A1.1.1, formerly known as IgG 50359, and sold by Strategic Diagnostics and the clone EW75C, generated by Dstl Porton Down, sold by BBI solutions. The mass spectrometric raw data can be found separately in the Supplementary Materials (ABID/input mass spectra).*

[2] Tscheuschner, G.; Schwaar, T.; Weller, M.G. Fast Confirmation of Antibody Identity by MALDI-TOF MS Fingerprints. Antibodies (Basel) 2020, 9, doi:10.3390/antib9020008.

Fingerprint raw data

The raw spectra and the fingerprint peaks of clones A1.1.1 and EW75C, according to [69] are located in the “\ABID\input spectra” or “\ABID\output” directory respectively.

Description of the semi-automated data evaluation with the python script

The data evaluation of the fluorescence signals was performed with Python 3.7 in the Spyder 3.2.2 by the Anaconda environment. The script supplied is intended for exactly this application. After the data has been captured as a sequence of .fits, raw images for the processing the script requires the following manual inputs: a "roiRegion.txt" file featuring a 50 x 50-pixel window around the approximate laser center position, a "hotpixel_1920_1200.txt" file, which includes all known hot pixels of the sensor and the approximate laser center position (S21) as starting guess for the fit.

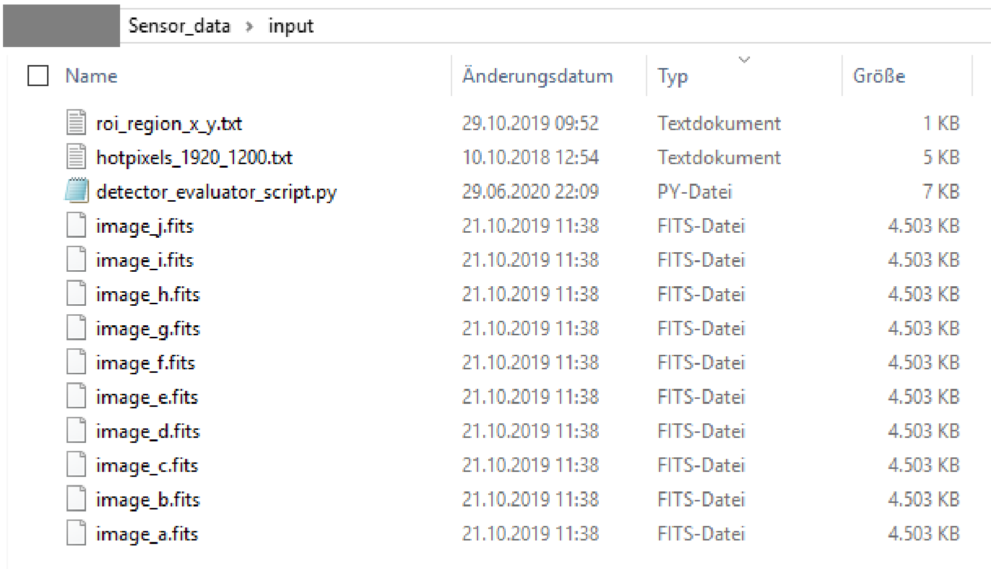

Figure S20. The "roiRegion.txt", the "hotpixel_1920_1200.txt", the raw data files and the detector_evaluator_script.py in the working directory. The result is in the “\Sensor_data\output” directory as "result.txt".

Determination of the coordinates of the laser point

In order to determine the approximate laser center coordinates for the first frame, a .fits file is opened with suitable software, and the coordinates are determined manually. To calculate the ROI[3] region, ±25 pixels around the approximate X- and Y- laser center pixels are entered in the "roiRegion.txt" file (S19).

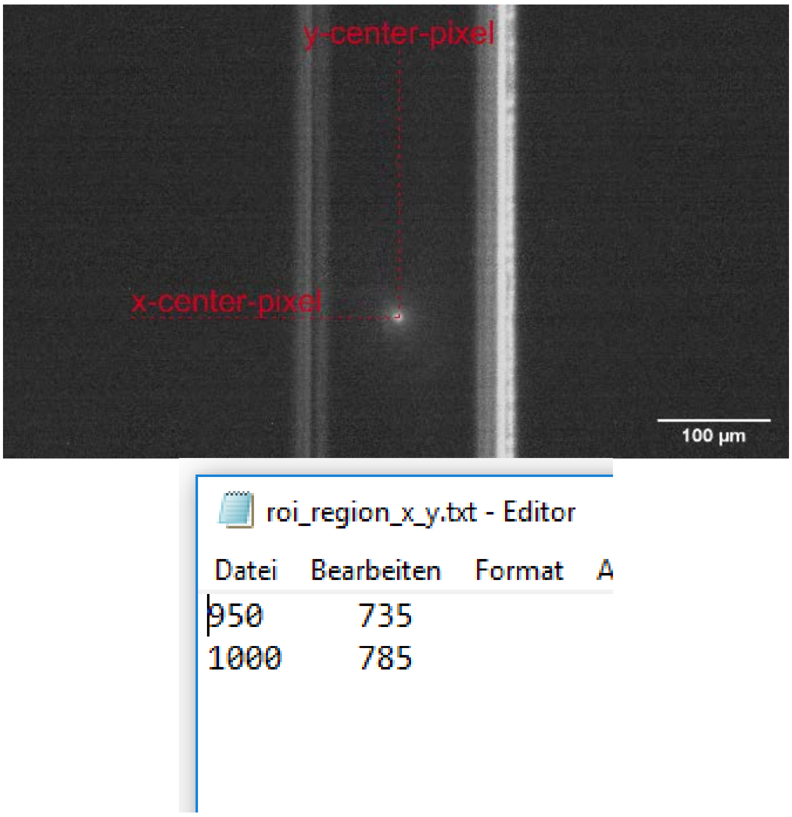

Figure S21. Approximate determination of the laser center (left) and definition of the ROI region in the "roiRegion.txt" file.

[3] Region of Interest

Finally, the chosen working directory must be copied in the script, and the manually determined laser center coordinates (S21) need to be entered as the first guess for the fit as "guess_prms" (S22). Now the script will save the intensities along with the correctly determined laser center coordinates as "results.txt" when executed.

```
#####################################################################################
# 1) Set the working directory:                                                     #
#####################################################################################

sourceDir = "C:\\Sensor_data\\input\\"

if not os.path.isdir(sourceDir):
    raise NotADirectoryError(sourceDir)

```

```

#guess_prms = [(x-center-pixel ,y-center-pixel ,4 ,4 ,1000 , 0)]
guess_prms = [(970 ,753 ,4 ,4 ,1000 , 0)]


```

Figure S22. Definition of the working directory with the raw files (top) and setting the x- and y-center coordinates as the first guess for the fit (bottom) the additional values of the guess represented the spot radius and the peak height and were found to be optimal for our settings.
